# Bipartite Functional Fractionation within the Default Network Supports Disparate Forms of Internally Oriented Cognition

**DOI:** 10.1093/cercor/bhaa130

**Published:** 2020-06-04

**Authors:** Rocco Chiou, Gina F Humphreys, Matthew A Lambon Ralph

**Affiliations:** MRC Cognition and Brain Sciences Unit, University of Cambridge, Cambridge UK

**Keywords:** connectivity, default-mode network, memory, semantic cognition, topography

## Abstract

Our understanding about the functionality of the brain’s default network (DN) has significantly evolved over the past decade. Whereas traditional views define this network based on its suspension/disengagement during task-oriented behavior, contemporary accounts have characterized various situations wherein the DN actively contributes to task performance. However, it is unclear how different task-contexts drive componential regions of the DN to coalesce into a unitary network and fractionate into different subnetworks. Here we report a compendium of evidence that provides answers to these questions. Across multiple analyses, we found a striking dyadic structure within the DN in terms of the profiles of task-triggered fMRI response and effective connectivity, significantly extending beyond previous inferences based on meta-analysis and resting-state activities. In this dichotomy, one subset of DN regions prefers mental activities “interfacing with” perceptible events, while the other subset prefers activities “detached from” perceptible events. While both show a common “aversion” to sensory-motoric activities, their differential preferences manifest a subdivision that sheds light upon the taxonomy of the brain’s memory systems. This dichotomy is consistent with proposals of a macroscale gradational structure spanning across the cerebrum. This gradient increases its representational complexity, from primitive sensory-motoric processing, through lexical-semantic representations, to elaborated self-generated thoughts.

## Introduction

The discovery of the brain’s default network (DN) epitomizes the serendipity of science. In the fledging period of human functional neuroimaging, researchers primarily employed externally oriented tasks (e.g., visual search, speech recognition, or finger tapping) to demarcate and catalogue brain regions responsive to external stimuli. The serendipitous discovery of the DN is consequent to using passive viewing (rest) as a contrasting baseline—some early studies observed that a set of brain regions, including the angular gyri and midline structures (medial prefrontal and posterior cingulate cortices), reliably exhibit heightened activity during passive moments yet become deactivated during active tasks (e.g., Andreasen et al. 1995). This task-negative resting-state activity was initially treated as an inadequacy of experimental design that failed to control for confounding variables (for discussion, see Buckner 2012). The turning point in the field’s conceptualization about the DN’s functionality came when Raichle et al. published their trailblazing work in 2001—rather than treating the DN’s activity as a failure of designing a proper baseline, Raichle et al. postulated that the task-negative regions collectively contribute to human cognition when the mind is disengaged from external tasks and returns to the “default mode” (rest) (Raichle et al. 2001). After their seminal work, subsequent research found that the ebb and flow of neural activities in DN regions tend to synchronize, forming a cohesively oscillating network (e.g., Greicius et al. 2003). It is now well-established that DN activity increases during various introspectively focused tasks (e.g., reminiscing the past, envisaging the future, reflecting on self, and empathizing with others; Kelley et al. 2002; Saxe and Kanwisher 2003; Spreng and Grady 2010). Subsequent research that combines fMRI and experience sampling (a behavioral protocol that intermittently probes thoughts) has provided more definitive measures linking the occurrence of subjective experiences (mind-wandering) with DN activity (Christoff et al. 2009). Recent research has further demonstrated that reliance on internally constructed representations (from brief memoranda kept in working memory to lengthy episodes of long-term memory) might be a crucial factor that regulates the degree of the DN’s involvement. For example, whereas DN activity is abated by decisions guided using immediately perceivable inputs, it is enhanced by decisions guided using contents of working memory (Murphy et al. 2018, 2019a). This argues against an oversimplified definition based on the DN’s absence during externally focused tasks and instead emphasizes its active participation in circumstances wherein task performance is sustained by internally constructed representations. Despite significant progress over the past decades, the complicated relationship between the DN and different types of introspective processes remains unclear. This background provides the milieu of the present investigation.

It is now apparent that the once prevailing “task-rest” dichotomy is inaccurate in describing the DN’s functionality. Updated frameworks have been proposed to accentuate its role in granting conscious access to memories and thoughts, untethering the human mind from the immediate here and now (e.g., Buckner and DiNicola 2019). However, we still lack an encompassing framework to account for its omnipresent involvement in different types of introspective activities, as well as the nuanced subdivision within this network in relation to different tasks. To tackle these challenges, various attempts have been made 1) to parcellate the brain into functionally distinct networks and 2) to uncover the cardinal organizing principles with which brain networks join forces or split up. For instance, based on the brain’s task-free intrinsic connectivity and hierarchical clustering, it has been shown that the whole-brain’s inherent connectivity fractionates into seven primary networks (Yeo et al. 2011; note that other schemes of parcellation have been used, resulting in different numbers of subnetworks), and the DN could be further partitioned into multiple subnetworks with them preferentially associated with different tasks (Andrews-Hanna et al. 2010). While such evidence demonstrates the heterogeneity of functional brain modules both at macroscale (whole-brain) and mesoscale (within the DN), it remains unclear whether there is a cardinal organizing dimension along which networks serving similar purposes unite while networks serving distinct purposes bifurcate. However, some recent studies offer clues that this organizing dimension exists and, more importantly, operates as a general principle at multiple levels. First, Margulies et al. showed that the whole-brain’s complex connectivity pattern can be condensed into a topographical polarity that explains a large proportion of cerebral layout, with regions serving outwardly oriented activities (perception and action) on one end of the spectrum and regions serving introspective activities on the other end (Margulies et al. 2016). A similar structure, albeit more rudimentary, is found in a marmoset monkey’s brain, suggesting a shared evolutionary origin (Buckner and Margulies 2019). Second, Vidaurre et al. investigated how resting-state activity unfolds in time and uncovered a systematic temporal structure that could be delineated as switching between two states—the propagation of neural activity tends to cycle within either the “outwardly leaning” sensorimotor network or the “inwardly leaning” default network, with sporadic transition between the two major networks (Vidaurre et al. 2017). Third, Dixon et al. showed that the frontoparietal control network fractionates into two major subsystems with each involved in different types of executive control—one is more closely aligned with sensorimotor regions and more active when attending to external stimuli, whereas the other is more coupled with the DN and more active when attending to internal thoughts (Dixon et al. 2018). In light of these recent discoveries, we asked a critical question—while the DN, as a whole, is more active for introspective processes, it remains unknown whether there exists a similar bipartite split within the DN, with one subsystem favoring more externally oriented thoughts/contexts (e.g., empathizing with other people) while the other preferring more internally oriented thoughts/contexts (e.g., contemplating about self).

The research literature of semantic cognition offers important clues as to how the DN might fractionate into subsystems. Semantic cognition refers to the high-order human capacity to comprehend the meaning of inputs (e.g., to understand text/speech, to recognize a corkscrew and its function) and to produce behavioral outputs according to meaning (e.g., correctly using a corkscrew to open a bottle by twisting it) (Lambon Ralph et al. 2017). It is a critical cognitive faculty that interfaces internal representations of meaning (all our pan-modality knowledge about the world) with external modalities (all the incoming auditory, visual, tactile signals that need to be mapped onto generalizable, modality-invariant concepts). Decades of research have shown that the two major aspects of semantic cognition—the ability to represent contents of semantic knowledge and the ability to maneuver semantic contents in appropriate ways—rely on different nodes of the frontotemporal semantic network (SN). Semantic knowledge per se is encoded primarily by the anterior temporal lobe (ATL) and various auxiliary regions (Chiou and Lambon Ralph 2019), whereas the ability to flexibly select and recombine semantic knowledge relies jointly on the inferior frontal gyrus (IFG) and posterior middle temporal gyrus (pMTG) (for reviews, see Jefferies 2013; Lambon Ralph 2014; Lambon Ralph et al. 2017). Intriguingly, previous research on default-mode processes often incorporates these SN regions—the ATL, IFG, and pMTG—as “add-on” prongs of the DN, while the primary focus is on the three “core” regions of the DN—the medial prefrontal cortex (mPFC), posterior cingulate cortex (PCC), and angular gyrus (AG) (e.g., Andrews-Hanna et al. 2010; Bzdok et al. 2013; Spreng et al. 2013). Incorporating the SN into the DN is due to their commonality: Just as the core DN nodes, the key SN nodes are situated within the cortical realm that is deactivated by externally directed non-semantic tasks (particularly the ATL, see Visser et al. 2010). Moreover, many semantic-related regions have been found to link robustly with a broad swathe of the DN during both task and resting-state (Bzdok et al. 2013; Jackson et al. 2016; Humphreys and Lambon Ralph 2017). There are, however, differences—it has been shown that while the ATL and AG are both deactivated by externally directed visuospatial tasks, they show opposite patterns for semantic processes, with ATL activity elevating while AG activity lowering for semantic tasks (Humphreys et al. 2015). Taken together, these commonalities and differences motivate our focus on exploring the relationship between the DN and SN, with the aim to understand the division within the broad DN territory and the neurocognitive dimension behind the fractionation.

In the present study, we report the findings of two fMRI experiments whereby we systemically manipulated two critical neurocognitive dimensions that regulate the DN’s activity: First, participants were either constrained by specified correspondences among external stimuli, semantic meaning, and reaction within a short timeframe (Experiment 1) or were allowed to let internal theme-guided thoughts roam with little time pressure (Experiment 2); the former context demands attention to the external entities (visual stimuli and effector reaction), whereas the latter emphasizes attention to internal thoughts. Second, cognitive operations were directed towards visuospatial processing of external meaningless stimuli (mental rotation or visual search), internal thoughts about self (reflecting on self-traits, or autobiographical memory), internal thoughts about others (reflecting on others’ traits, or mentalization), as well as task-free mind-wandering (rest). To pre-empt the main findings, with these systematic manipulations, we found a clear bipartite split within the DN. While the broad DN, as a whole, strongly prefers introspective activity to perception and action, it fractionates functionally into two bodies—one system favors *self*-referential thoughts, and its activity *dwindles* when thoughts pertain to external entities. By contrast, the other system favors *other*-referential thoughts, and its activity *intensifies* when thoughts pertain to external entities. Moreover, neural dynamics within and between the two subsystems alter according to whether the task-contexts accentuate external stimuli or internal thoughts, providing further support to the bipartite structure. These findings are discussed with reference to recent proposals about the macroscale transition from unimodal/sensorimotor to transmodal/abstract zones that spans across the human cerebrum.

## Methods

### Participants

Twenty-four volunteers gave informed consent before their participation. The sample consisted of an 18/6 female-to-male ratio, with average age = 25 years old and standard deviation (SD) = 7. All volunteers are right-handed and speak English as their mother tongue. All of them completed the magnetic resonance imaging safety screening questionnaire before the experiment and reported not having any neurological or psychiatric condition. This study was reviewed and approved by the local research ethics committee.

### Design

Participants completed two experiments in a single session. In Experiment 1, we modified a well-established experimental paradigm that has been widely used to probe the neural basis of self-knowledge (e.g., Kelley et al. 2002; Krienen et al. 2010; Meyer and Lieberman 2018). Participants were asked to complete various tasks under a typical psychophysical context in which they were required to make a trial-by-trial response as quickly as possible within a brief timeframe. There were four conditions: 1) the self-referential task: participants read adjectives describing various personality traits and assessed whether the words suitably describe the characteristics of themselves. 2) The other-referential task: similar to the self-referential task, participants read adjectives and assessed whether the depictions suit the Queen Elizabeth II’s personality. 3) The visuospatial task: participants viewed a pair of meaningless scrambled visual patterns and answered whether the two were mirror inverse of each other. 4) Mind-wandering (rest): participants passively viewed a blank screen while awaiting the next task block to begin.

Experiment 1 consisted of three runs of scanning. Stimuli were presented using a block design, controlled with E-Prime (Psychology Software Tools). Each run was 432 s in duration, with each of the four conditions (self/other/visuospatial/rest) having six blocks. The order in which task conditions were presented was fully counterbalanced across participants so that each task condition was equally likely to appear in every possible slot of the sequences, with stimuli randomly drawn from a designated stimuli set (also counterbalanced across participants) for a given scan and shuffled across blocks. Each block contained five trials. Each trial began with a fixation dot (0.8 s), followed by visual stimuli shown for 2.8 s and no intertrial interval. In the self and other conditions (see the Supplementary Figure 1 for task procedure), we displayed the target of assessment (self or queen) above the fixation dot and asked participants to answer whether an adjective word (below the central dot) suitably described the personality of the target individual. In the visuospatial condition, we displayed two scrambled visual patterns (squiggly lines made from randomly breaking and recombining word text of other two conditions); participants performed mental rotation and answered whether the two patterns were left/right flipped. Participants were required to react as fast (and accurately for the visuospatial task) as possible within the 2.8-s limit. In the rest periods, we displayed a blank screen and instructed participants to stay awake and still while awaiting the next task. Participants reacted to the questions by pressing a button on a MR-compatible response pad with their right hand. All visual stimuli were displayed on a mid-gray background, using a high-resolution LCD goggles (NordicNeuroLab) mounted on top of the head coil.

A total of 180 adjectives were used in the self and other conditions, with 90 words used in each condition. The mapping with which a word was shown in either the self or other condition was fully counterbalanced across the participants so that each adjective was equally probable to be assessed with reference with self or the queen. The stimuli sets contained equal proportion of positive traits and negative traits, evenly allocated to the two conditions. Moreover, we controlled the lexical frequency (based on the British National Corpus) and word length (based on the number of letters; average ± SD: 8 ± 2 letters in both conditions) of the stimuli so that the stimuli sets used in the two task conditions were matched on these psycholinguistic properties.

Experiment 2 consisted of four runs of scanning. We adopted and modified an established experimental design of a landmark study that has been widely used to probe functions of the default network (Spreng and Grady 2010). Stimuli were presented using a slow event-related design. Each run was 432 s in duration. There were four conditions—autobiographical memory (AM), theory of mind (ToM), visuospatial search (VS), and rest. Each run consisted of 18 active task events (AM, ToM, VS; 6 events per condition per run, giving 24 events per condition for the whole experiment) and 18 randomly jittered rest intervals intervening between active task events (duration of jittered rest—average ± SD = 6 ± 2.74 s, range: 2–12.5 s). The order in which task conditions were presented was fully counterbalanced across participants so that the events of each task condition were equally likely to appear in every possible position of the sequences, with stimuli randomly drawn from a designated stimuli set (also counterbalanced, hence each set being equiprobable to be used in runs 1–4). In the AM task, participants were required to recollect personal experiences (including their own thoughts and feelings at the time, as well as the temporal–spatial contexts) related to the theme of a photograph depicting human activity (see below for details). In the ToM task, participants were required to imagine how the individual(s) in the photograph might be thinking or feeling. In the VS task, participants viewed mosaic scrambled patterns and search for a tiny gray triangle hidden in the patterns.

Each trial began with a cue word for 1 s, prompting participants the upcoming task (AM, remember; ToM, imagine; VS, search). This was followed by a centrally presented image (600 × 380 pixels), as well as a short passage below the image, displayed for 15 s. In the AM and ToM conditions, the images were photographs depicting people in various situations of life (e.g., seeing a dentist, cooking in the kitchen, protesting in a rally, etc.). The short text below was 20 words in length, serving as a cue to help participants with autobiographical recollection or imagining about others’ thoughts and feelings (e.g., AM: “Remember the time you learnt the outcome of Brexit referendum. How did you feel? How did you respond to it?” or ToM: “Imagine what the girl who’s holding the Christmas cracker is thinking and feeling. Also imagine how her grandpa would respond”). In the VS condition, the images were made from scrambling images of other conditions into random mosaic patterns, and the text below simply said “Is there a tiny triangle hidden in the pattern?” The triangle was present in a half of the trials. After the 15-s interval during which participants recollected, imagined, or searched, a question was shown for 2 s asking participants to rate how vivid their memory or imagery was (1, very vivid; 2, somewhat vivid; 3, not at all vivid) by pressing a designated button on the response pad. In the VS condition, the question asked if there was a triangle in the pattern, and participants answered with a binary “yes” or “no” key response. Participants were instructed to concentrate on the recollection, imagery, and visual search during the 15-s interval, and they should make a response only when the question was shown at the end.

Prior to scanning, we used a similar three-step training protocol to that of the Spreng and Grady study (Spreng and Grady 2010) to ensure that all of the participants were able to engage confidently in retrieving autobiographical events related to the picture’s topic and imagining the thoughts and feelings that people in the picture might have. Forty-eight photographs depicting human activities and interactions, all of them containing at least one person or more, were used in the AM and ToM conditions, with a half of them used in one condition and the remaining half used in the other. We fully counterbalanced the images and conditions across participants to ensure that 1) each photograph was equally likely to be presented as a cue in the AM and ToM condition, ruling out stimulus-specific effects, and, 2) for each participant, separate sets of photographs were used as cues for the AM and ToM contexts, thus preventing contamination from the other condition.

### Scanning

Full details of data acquisition parameters, procedures of preprocessing, and statistical analysis are reported in Supplementary Material. Here we provide the key information: The brain regions of our primary interest are situated in the rostro-ventral aspects of the brain (e.g., the ATL, the vmPFC), which are known as particularly susceptible to signal dropout (Halai et al. 2014). To combat the dropout issue in these target areas, we adopted a dual-echo EPI sequence, which has been demonstrated to improve signal-to-noise ratio around these rostro-ventral regions relative to other conventional imaging protocols (e.g., Halai et al. 2014; Humphreys et al. 2015; Chiou et al. 2018). A customized procedure was used to combine the two echo time images of each brain volume. Using SPM8, we integrated the standard preprocessing procedures with multiple correction methods to prevent image distortion and improve interparticipant alignment for group analysis. For statistical analysis at the individual level, all of experimental conditions of each experiment were modeled explicitly as separate regressors, while task-free resting periods (rest) were modeled implicitly, as per the default of SPM. Blocks/events corresponding to our experimental factors were convolved with a canonical hemodynamic response function. Response execution periods in Experiment 2 were modeled as a separate regressor. Six motion parameters were entered into the model as covariates of no interest. Behavioral reaction times were also modeled as parametric modulators to rule out the confounding influences due to task difficulty or cognitive effort.

### Regions of Interest (ROIs)

In Supplementary Material, we report the full details of the 10 ROI coordinates in the MNI stereotaxic space and pinpoint the sites on the template images. All of these ROIs are selected independent of the task contrasts of the two experiments, based on the meta-analysis outcomes of relevant literatures. Specifically, we surveyed four relevant studies of neuroimaging meta-analyses (Bzdok et al. 2012; Noonan et al. 2013; Humphreys and Lambon Ralph 2014; Rice et al. 2015) about the neural correlates of default-mode functions (the targets include brain area robustly related to “mind-wandering, daydreaming, self-representation, autobiographical memory, theory of mind, episodic memory”), semantic cognition (including “semantic memory, semantic control, conceptual knowledge”), and social cognition (including “theory of mind, mentalizing, empathy”). Using the activation likelihood estimates (ALE) identified by these topic-focused meta-analyses, we identified 10 important nodes closely associated with different aspects of default-mode and semantic-related functions—the dmPFC, vmPFC, PCC, bilateral AG, bilateral TPJ, left ATL, left IFG, and left pMTG. At each of the ALE peak coordinates, we created a spherical ROI with 6-mm radius. Next, we evaluated the suitability of the selected ROIs by using the “association test” function of NeuroSynth (a large-scale fMRI meta-analysis platform; Yarkoni et al. 2011). Based on the results of term-based search (“default-mode, semantic memory, theory of mind, mentalising”) that contain 2214 studies and 84 573 peaks, NeuroSynth generated the “association test” maps that represent brain areas statistically significantly (FDR corrected at *q* < 0.01) associated with these terms. We then compared our selected ROIs and these maps and confirmed that all of the 10 chosen ROIs are encompassed within the meta-analytic maps (see the Supplementary Figure 1 for illustrations).

### Dynamic Causal Modeling (DCM)

We performed a series of six DCM analyses using DCM10 in SPM8 (Friston et al. 2003). DCM-1, DCM-3, and DCM-5 were performed on the data of Experiment 1, whereas DCM-2, DCM-4, and DCM-6 were done on the data of Experiment 2. For each DCM, we localized the network nodes based on the same coordinates that we defined using relevant literatures, verified using NeuroSynth, and confirmed their involvement in the tasks using a series of ROI analysis. Each network node was localized using a spherical ROI with 6-mm radius centered at the designated coordinate. Activated voxels within each node were identified using the relevant contrasts (i.e., Experiment 1: Self + Other > Rest; Experiment 2: AM + ToM > Rest) thresholded at *P* < 0.001 for voxel intensity. We used the SPM’s default algorithms to extract the first eigenvariate to obtain the time series of activity, as per the standard procedure of DCM. This process was repeated for all of the selected regions in each participant. All of the DCM were set to have one state per region and without stochastic modulatory effect. Based on the extracted activity, we constructed and estimated DCM separately for each participant and then employed the variational Bayesian analysis (VBA) toolbox (Daunizeau et al. 2014; Rigoux et al. 2014) to conduct random-effect (RFX) group analyses using its functions of Bayesian model selection. The RFX procedure outputted the protected exceedance probability (PEP; Rigoux et al. 2014), which is a unbiased probabilistic estimate that quantifies how likely that, for an individual randomly drawn from the population, the model being considered provides the best fit to this person’s fMRI data than any other provided model, above and beyond the chance level. The chance level represents the probability of all models being equally likely (1/*K*, with *K* being the number of models; we had 0.33 as the chance level for all analyses). Bayesian model selection does not rely on a binary, arbitrary cutoff threshold. Rather, it relies on identifying the model that shows the highest, sufficiently above-chance PEP.

Here we specify the model spaces. 1) In DCM-1 and DCM-2, we focused on the relationship between the dmPFC and the vmPFC; for each DCM, we constructed three models, and each model contained two nodes, with the input signal entering via the dmPFC, the vmPFC, or both nodes. The two nodes were set to be mutually connected. 2) In DCM-3 and DCM-4, we focused on the relationship among the IFG, the ATL, and the vmPFC; for each DCM, we constructed three models, and each model contained three nodes, with the input signal entering via the IFG, the ATL, or the vmPFC. All of the three nodes were set to be mutually connected. 3) In DCM-5, we focused on the relationship between nodes within the SN (the IFG, the pMTG, the dmPFC, and the ATL) and tested only using Experiment 1’s data. We constructed three models, and each model contained four nodes, with the input signal entering via the IFG, the pMTG, or the dmPFC. Here we did not include the ATL as an entry node because the outcome of the preceding DCM-3 had already indicated that the probability of ATL-input model being the winner was close to zero. In order to constrain the size of model space to having three models and maintain a consistent chance level across analyses, we included only the IFG-, pMTG-, and dmPFC-input models. The four nodes were set to be mutually connected. 4) In DCM-6, we focused on the relationship between nodes within the core DN (the PCC, the AG, the vmPFC) and tested only using Experiment 2’s data; we constructed three models, and each model contained three nodes, with the input signal entering via the PCC, the AG, or the vmPFC. All of the three nodes were set to be mutually connected.

## Results

Experiment 1 created a restrictive and relatively outwardly directed context—participants read text about personality traits, assessed its pertinence with regard to either self or another individual, and reacted with a button-press within a short timeframe using specified rules about the mapping between meanings and fingers. By contrast, Experiment 2 created a less restrictive and inwardly directed context—prompted by a photograph and its theme, participants either recalled autobiographical memory (AM) related to the theme or imagined how the people in the picture might be thinking or feeling (theory of mind/ToM). No restriction on thinking, no requirement for explicit reaction, and little time pressure were imposed while participants engaged in AM and ToM, allowing immersion in introspective activates. These contrastive task settings allowed investigations into whether the brain responds differentially to internally versus externally directed situations. Analysis of behavior data showed that conditions designed to probe the functions of DN and SN are matched on their psychophysical profiles (see Supplementary Material). Analyses of fMRI data were performed at multiple scales. We first interrogated the entire brain to examine whether different brain structures exhibit preferences for different contexts. We subsequently zoomed in on the regions of interest (ROIs) within the DN and SN, comparing the two networks’ preferential reactions to different task settings. In addition to the direct contrast between DN and SN nodes, we focused specifically on the functional subdivision at a provincial scale, inspecting the responses of adjacent areas within the mPFC and within the inferior parietal lobule (IPL). This local-scale inspection was motivated by recent mounting evidence that 1) the broad mPFC zone harbors two subsections, with its dorsomedial part (the dmPFC) being more connected with the SN and its ventromedial part (the vmPFC) being more connected with the DN (Bzdok et al. 2013), and that 2) a similar bisection has also been observed in the IPL, with its anterior section (the temporoparietal junction/TPJ) being more connected with the SN and posterior section (the AG) more connected with other DN nodes (Uddin et al. 2010; Mars et al. 2011; Braga et al. 2019).

**Figure 1:**
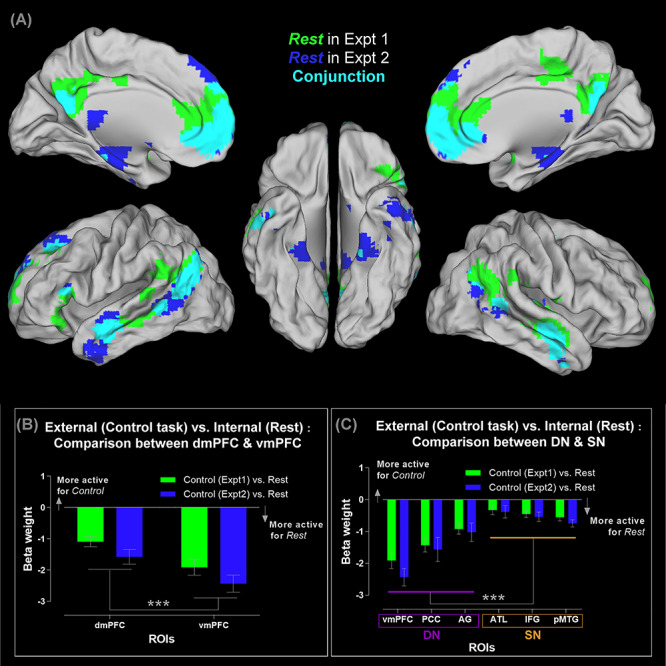
(*A*) Brain regions showing significantly greater activity for mind-wandering (rest), compared to the visuospatial control task, thresholded at *P* < 0.05 (FWE-corrected for whole-brain voxel intensity). Significant clusters in Experiment 1 (green), Experiment 2 (blue), and their conjunctions (cyan). (*B*) ROI analysis of the dmPFC versus vmPFC. Note that negative β-weight means more active for mind-wandering (rest), compared to the visuospatial control tasks. (*C*) ROI analysis of the three core DN areas (the vmPFC, PCC, and AG), as well as the three core SN areas (the ATL, IFG, and pMTG). See Supplementary Material for the locations of ROIs that are rendered on a template brain. ^***^P < 0.001.

### Resting-State/Task-Negative Activity Reveals a Bipartite Split between the DN and SN

The traditional definition of the default system is a set of brain regions whose activity drops when the mind is engaged by goal-driven behavior yet rises when the mind is idle during wakeful resting state. While the DN and SN both fall within the realm of this purported “task-negative” zone, it remains unclear whether they respond differentially to resting-state wherein the mind is free from the preoccupation of explicit goals and salient stimuli. To this end, we included, in both experiments, visuospatial control tasks that are designed to elicit deactivation of the classic “task-negative” zones—in Experiment 1, participants mentally rotated squiggly stimuli and compared their visual configurations, while in Experiment 2 they searched for a tiny triangle hidden in mosaic patterns. We began by a whole-brain interrogation to identify regions that are significantly more active during rest, compared to the visuospatial conditions, stringently thresholded using FWE correction (*P* < 0.05) for whole-brain voxel-wise intensity. Results corroborate the classic definition of the “task-negative” neuroanatomy: As shown in Figure 1*A*, resting-state activity is amplified in extensive swathes of the DN and the SN, relative to the visuospatial tasks of mental rotation and visual search. As illustrated by the conjunctive clusters of Figure 1*A*, the peak points of resting-state activation concur between the two experiments at the key nodes of DN (the mPFC, PCC, and left AG), as well as the key nodes of SN (the bilateral ATL, left IFG, and bilateral pMTG). Importantly, the two networks differ in their magnitude of resting-state activation. As revealed by the ROI analysis, compared to SN regions, brain regions conventionally classified as core constituents of the DN exhibit reliably greater responses to resting state (i.e., greater extent of deactivation for the visuospatial tasks). This is observed both at the regional and multinode network levels. At a local level (within the mPFC), resting state triggers significantly greater activity of the vmPFC (a subregion more closely linked with other DN regions) compared to the dmPFC (a subregion more closely linked with the SN), resulting in reliable effects seen in both experiments (*F*_(1,23)_ = 13.16, *P* = 0.001, ${\eta}_{\mathrm{p}}^2$ = 0.36; see Fig. 1*B*). At a network level (the broad “task-negative” zones), the three ROIs known to form the core DN (the vmPFC, PCC, and AG; see Andrews-Hanna et al. 2010) show significantly greater activity during resting state, relative to the three ROIs known to form the core SN (the ATL, IFG, and pMTG; see Lambon Ralph et al. 2017), also reliably found in both experiments (*F*_(1,23)_ = 56.20, *P* < 0.001, ${\eta}_{\mathrm{p}}^2$ = 0.71; see Fig. 1*C*). Taken together, despite the fact that the DN and SN show common tendencies (i.e., activity heightens during rest and lessens during visuospatial tasks), activity of DN regions during rest is significantly more intensive, dwarfing the comparatively moderate response of SN regions. Moreover, our results indicate that while the wide mPFC is sometimes conflated as a unitary, monolithic region, it is actually heterogeneous, with its ventral section leaning towards the DN and responding vigorously during resting state and its dorsal section leaning towards the SN and responding moderately.

**Figure 2:**
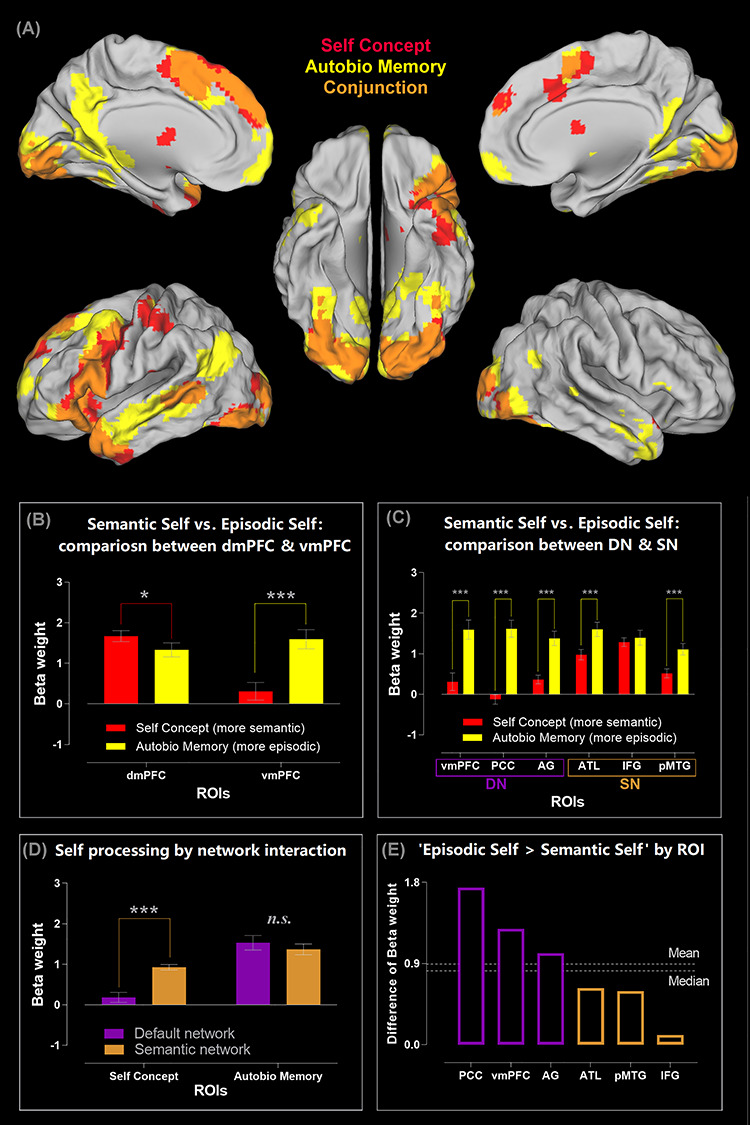
(*A*) Brain regions showing significantly greater activity for the two types of self-related tasks: The self-concept evaluation task (red), for the autobiographical memory task (yellow), and their conjunctions (orange). All contrasts were against the baseline of mind-wandering (rest), thresholded at *P* < 0.05 (FWE-corrected for whole-brain voxel intensity). (*B*) ROI analysis reveals an interaction between brain regions (dmPFC vs. vmPFC) and the types of self-related tasks (self-concept vs. autobiographical memory). (*C*) ROI analysis of the three core DN areas (the vmPFC, PCC, and AG), as well as the three core SN areas (the ATL, IFG, and pMTG). (*D*) The significant interaction between the two networks and the two types of self-processing. (*E*) The six ROIs ranked by their preference for autobiographical memory over self-concept, with all of the three DN regions showing a noticeably greater preference than all of the three SN regions. ^***^P < 0.001, ^*^P < 0.05.

A close inspection on the results of whole-brain analysis (Fig. 1*A*) revealed some discrepancy between the resting-state activities of Experiments 1 and 2 alongside their commonality. While resting-state activity of Experiments 1 and 2 occupied the typical neural estates of DN and SN with considerable overlaps (the cyan clusters, Fig. 1*A*), the resting periods of each experiment elicited specific regions that were less involved during the resting time of the other experiment (the green and blue clusters, Fig. 1*A*). The resting times of Experiment 1 preferentially engaged the dorsal section of the PCC and the TPJ, while those of Experiment 2 preferentially engaged the medial temporal lobe/MTL (the parahippocampal cortex/PHC, and the entorhinal/perirhinal cortices). Intriguingly, this finding fits with the separation of “Network-A” and “Network-B” discovered in recent resting-state studies (*cf.*, the original nomenclature; Braga and Buckner 2017; Braga et al. 2019)—while Network-A and Network-B are closely juxtaposed, they dissociate on several “diagnostic” areas, with Network-A preferentially including the PHC and Network-B preferentially encompassing the dorsal PCC and TPJ. A potential contributing factor to the pattern that we found might be the differences in task settings that biased subsequent resting-state activity. While the resting intervals in both experiments were yoked in terms of psychophysical settings (i.e., participants passively viewed a fixation cross without performing any task), the two experiments differed in the task contexts that preceded a resting period. Specifically, Experiment 1 demanded trial-by-trial semantic judgment on personality traits, whereas Experiment 2 provided a response-free context allowing detailed mnemonic retrieval to unfold. There has been reliable evidence showing that task settings can modulate subsequent resting-state activation and connectivity (e.g., performing personality judgment selectively enhances subsequent connectivity of DN/SN regions during rest; see Meyer et al. 2019). This hints that an episode of goal-directed mental activity might shape subsequent network architecture during rest, be the taxonomy of networks being “Network-A versus Network-B” or “DN versus SN.” Future research is needed to ascertain the cause and effect (e.g., autobiographical memory might have lingering effects that preferentially boost the resting-state activity of PHC/MTL/Network-A, whereas sociosemantic judgments might preferentially boost the TPJ/dorsal PCC/Network-B). Additional information about the comparison between resting state and control tasks across experiments is reported in Supplementary Material (see the Supplementary Figure 2).

### Different Types of Self-Referential Mental Activities Reveal a Bipartite Split between the DN and SN

Mental activities related to “self” are intuitively “inwardly leaning” and have been known to be strongly associated with the DN (for review, see Qin and Northoff 2011; Molnar-Szakacs and Uddin 2013). In particular, much emphasis has been laid on the mPFC—various types of self-related mental activities robustly engage this area, and pathology of the mPFC leads to difficulty in self-regulation (for review, see Wagner et al. 2012). Previous neuroimaging investigation into the neural basis of self-related processes has adopted two primary types of experimental approaches. One pervasive approach is asking participants to access self-concept via evaluating adjectives about personality traits with reference to self, which entails participants processing semantic information, bearing the task rules in mind, and reacting on a trial-by-trial basis within a time limit (e.g., Kelley et al. 2002; Meyer and Lieberman 2018; Murphy et al. 2019b). The other popular approach is asking participants to recollect autobiographical memories related to a provided topic while participants have sufficient time (typically an interval longer than 10 s) to engage in mnemonic retrieval (e.g., Spreng and Grady 2010). The former approach is more “semantic” in nature and entails an outwardly focused context (reading text, pushing buttons), whereas the latter is more “episodic” in nature and entails an inwardly focused context. Our experiments provided us a unique window into how the DN and SN might respond differently to self-related activities under these two types of situations. We began unpicking this difference by examining the whole-brain activity pattern, comparing the distribution of activities induced by the self-trait assessment task (“semantic-self”) and the autobiographical recollection task (“episodic-self”). As illustrated in Figure 2*A*, both tasks significantly increase activities of various DN and SN regions compared to rest, replicating previous data that goal-directed introspective activities heighten the DN compared to the resting state wherein aimless mind-wandering often happens (Spreng and Grady 2010). However, closer inspection on the pattern reveals that while expansive swathes of the SN regions are recruited by both tasks, the three core DN nodes (the vmPFC, PCC, and AG) are exclusively recruited only when one processes “episodic-self” during the autobiographical task. Such differential engagement of the DN and SN by the two types of self-related processing becomes clearly manifested when we examine the ROIs (note that all of the contrast baseline here is resting-state, rather than external visuospatial tasks, hence allowing us to assess whether goal-directed introspective activity further enhances neural reaction on top of resting-state activity, without the contamination from suppressive effects of external tasks): As Figure 2*B* shows, within the mPFC, its “SN-leaning” dorsal section is more active for self-concept, whereas its “DN-leaning” ventral section is more active for autobiographical memory, resulting in a significant interaction (*F*_(1,23)_ = 29.37, *P* < 0.001, ${\eta}_{\mathrm{p}}^2$ = 0.56). This dissociation is not only found at the local scale within the mPFC but also seen at a more global scale: As Figure 2*C* illustrates, while nearly all of the ROIs exhibit preference for autobiographical memory over self-concept, this preference is evidently much attenuated in the three ROIs belonging to the SN, particularly in the inferior frontal gyrus (*F*_(1,23)_ = 19.72, *P* < 0.001, ${\eta}_{\mathrm{p}}^2$ = 0.46). To dissect this network-by-task interaction further, we separately scrutinized the neural response to each task and found that retrieval of autobiographical memory engages regions of the DN and SN to an equivalent extent (*P* > 0.32, Fig. 2*D* right). By stark contrast, accessing self-concept primarily recruits the SN yet minimally engages the DN, leading to a significant difference (*P* < 0.001, Fig. 2*D* left). Finally, we plotted the magnitude of preference for autobiographical memory over self-concept (indexed as their β-weight difference) for each ROI to manifest their relationship: As Figure 2*E* shows, while all of the ROIs are more active for autobiographical recollection, there is a clear split, with all of the DN nodes exhibiting strong preference above the average and median level (driven primarily by their disengagement during the self-concept task) and all of the SN nodes showing much mitigated preference falling below the average and median (indicating the SN’s all-embracing participation in both tasks). These results clearly indicate a bipartite split in the neurocognitive substrates for different types of self-related processing—accessing self-related semantic knowledge relies on the SN while barely involves the DN, whereas accessing episodic memory of life events is a multifaceted process that fuses temporospatial details with semantic meaning and is supported by both the DN and SN.

### The Choice of Baseline Affects whether mPFC Activation Appears in the Contrast of Task Situations

**Figure 3:**
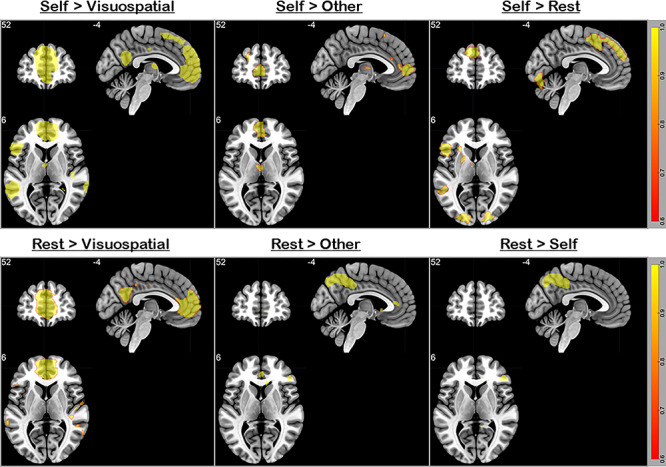
All contrasts were thresholded at *P* < 0.05 (FWE-corrected for whole-brain voxels). The upper row illustrates the contrast of (left to right) “self-knowledge > visuospatial task,” “self-knowledge > other-referential knowledge,” and “self-knowledge > resting-state baseline.” Note that the choice of baseline affects the size of vmPFC cluster, with the rest-baseline engaging the vmPFC most, the visuospatial-baseline engaging the vmPFC least, and the other-baseline being intermediate. A highly similar pattern is found in the contrast of (lower row, left to right) “Rest > Visuospatial,” “Rest > Other,” and “Rest > Self,” indicating that mind-wandering (rest) equally engages the vmPFC as self-knowledge. The image follows the neurological convention—the left/right side on the image represents the left/right hemisphere.

Our results show that the dmPFC (also the vmPFC, albeit to a less extent) augments its activity when participants evaluate personality descriptions with reference to self, replicating previous findings (e.g., Kelley et al. 2002; Macrae et al. 2004; Heatherton et al. 2006). However, a closer inspection of previous studies reveals that this is actually driven by “less deactivation” for the “Self > Rest” contrast (which often results in no difference), compared to other contrasts that induce “more deactivation” due to greater mPFC activity during rest (e.g., “Other > Rest” or “Letter-case > Rest”). In light of such observations, we further inspected the data of Experiment 1 by comparing the self condition with three different baselines (other, visuospatial, and rest). All of the analyses were conducted using whole-brain interrogation, thresholded at *P* < 0.05 FWE-corrected for voxel-wise intensity. As illustrated in Figure 3, the vmPFC responds to the self-concept task and resting state with comparable activation level, resulting in no significant cluster in the vmPFC region in both contrasts (“Self > Rest” and “Rest > Self”; Fig. 3 right). This is consistent with previous findings that participants tend to think about themselves during the resting period (Meyer and Lieberman 2018), driving vmPFC activity to persist despite no task during rest. However, a significantly active vmPFC cluster emerges when we searched for “Self > Other” and “Rest > Other” (Fig. 3 middle, although the cluster size of “Rest > Other” is understandably smaller). The size of this cluster further expands when we searched for “Self > Visuospatial” and “Rest > Visuospatial” (Fig. 3 left). This suggests a graded participation—the vmPFC is least involved during the visuospatial task, most involved during the self-concept task; situated in between are the contexts of resting-state and the other-concept task. These results supplement what we report in the section above—when the reference of contrast is a passive rest interval, the autobiographical task (active, goal-directed processes while allowing immersion into self-generated thoughts) boosts vmPFC activity beyond its level during resting-state, whereas the self-concept assessment task (which requires attention to stimuli and output-effector) induces vmPFC activity that is just commensurate with its resting-state level.

### Self- versus Other-Referential Mental Activities Reveal a Bipartite Split between the DN and SN

**Figure 4:**
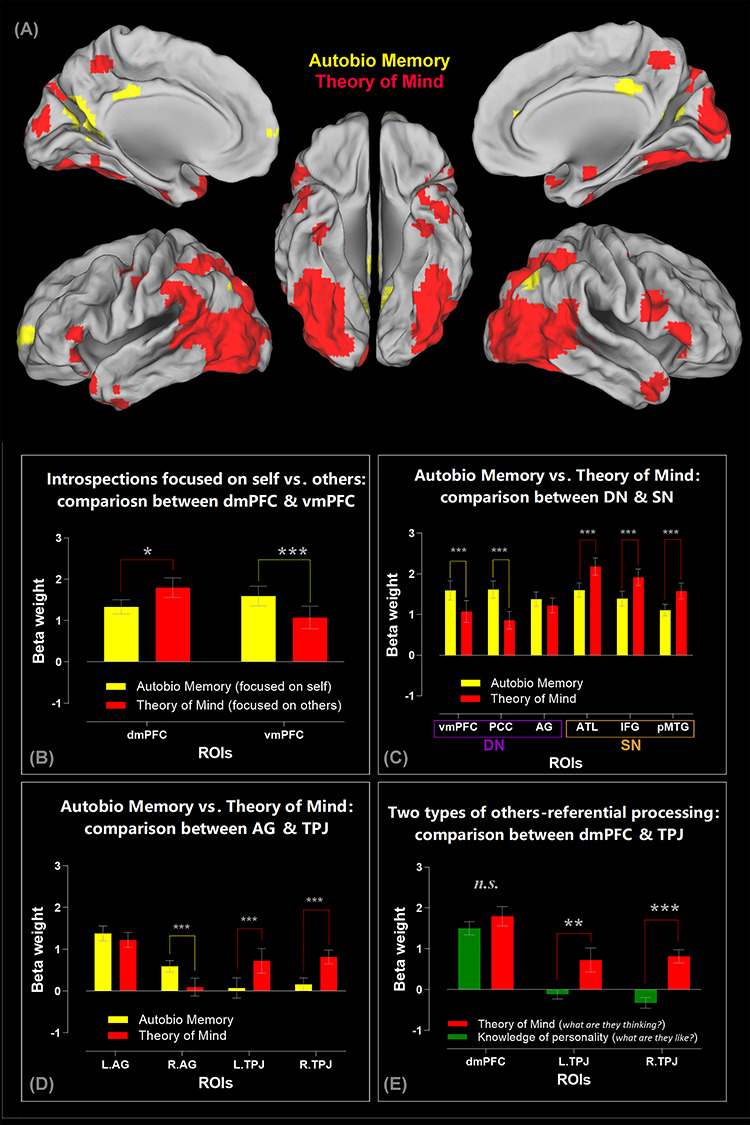
(*A*) A direct comparison between the two introspective tasks reveals the regions showing significantly greater activity for the autobiographical task (yellow clusters: Autobiographical Memory > Theory of Mind) and for the mentalizing task (red clusters: Theory of Mind > Autobiographical Memory). Statistics are thresholded at thresholded at *P* < 0.05 (FWE-corrected for whole-brain voxel intensity). In addition to the key finding that we elaborate in the main text (i.e., greater DN activity during autobiographical memory vs. greater SN activity during theory of mind), it is noteworthy that, in the mentalizing task, participants simulate the thoughts/feelings of the protagonist in the photograph while viewing/interpreting the semantic meaning of the visual scene. This is a multifaceted operation that entails visual attention and scene recognition, semantic interpretation, and theory of mind. Our results—namely, the red clusters—reflect this multifaceted nature: Relative to the autobiographical task, the mentalizing task imposes greater demand on visual processing, hence greater activation of the posterior vision- and attention-related cortices. (*B*) ROI analysis reveals an interaction between brain regions (dmPFC vs. vmPFC) and introspective tasks (autobiographical memory vs. theory of mind). (*C*) ROI analysis of the three core DN areas (the vmPFC, PCC, and AG), as well as the three core SN areas (the ATL, IFG, and pMTG), revealing a significant interaction between networks and tasks. (*D*) The significant interaction between the two inferior parietal subregions (the AG vs. TPJ) and the two types of introspective processing. (*E*) The significant interaction between the regions preferring other-referential tasks (the dmPFC, left TPJ, and right TPJ) and the two types of other-referential processes. Note that all of the ROI analyses here are based on β-weights compared against resting baseline. ^***^P < 0.001, ^**^P < 0.01, ^*^P < 0.05.

Decades of research has accumulated a myriad of evidence for shared neural substrates for understanding the mental states of both self and others (van der Meer et al. 2010; Molenberghs et al. 2016; Heleven and Van Overwalle 2018). This shared neurocognitive system for both self- and other-referential processing comprises the cortical midline structures (i.e., the dmPFC, vmPFC, and PCC) and some lateral regions (including the ATL, IFG, pMTG, and IPL). However, it remains unclear whether the constituent regions in this system split into subsystems depending on whether they prefer inwardly (e.g., reflecting on self) versus outwardly directed (e.g., mentalizing about others) processes. Experiment 2 provided conducive circumstances to study whether there is a schism due to preferred processes—during the AM situation, participants recollected their own memories/feelings about specific episodes of life, whereas during the ToM situation, they made inferences about the mental states of other people. While the two tasks differed on the focus of social target (inward/self vs. outward/others), they were yoked and matched by our experimental design: Identical, counterbalanced stimuli were used in both conditions to rule out stimulus-related effects, and no external response was required during the AM and ToM periods to encourage them to concentrate on introspective experiences during the lengthy interval. Results of the whole-brain search, based on the direct contrast between the AM and ToM contexts, reveal a bipartite split that corroborates our speculation: As shown in Figure 4*A*, all of the three core DN regions (the vmPFC, PCC, and AG) were preferentially more engaged by AM compared to ToM. By contrast, extensive stretches of the SN regions (including the three core SN nodes—the bilateral IFG, ATL, and pMTG—as well as regions related to ToM, such as the temporoparietal junction/TPJ) were more engaged by ToM than AM. Analysis of ROIs further highlights the granularity of such bipartite structure (like our previous analysis, all of the ROI analysis here is based on β-weight compared against rest, allowing us to gauge whether task-driven activity surpasses the baseline): As shown in Figure 4*B*, an evident split is found within the mPFC, with its “SN-leaning” dorsal section preferring other-referential/ToM processes and its “DN-leaning” ventral section preferring self-referential/AM processes (*F*_(1,23)_ = 15.87, *P* = 0.001, ${\eta}_{\mathrm{p}}^2$ = 0.41). The split is also observed at the network level (Fig. 4*C*), with the core DN midline structures (i.e., the vmPFC and PCC) exhibiting a strong preference for self- over other-referential processes and all of the three SN nodes (the IFG, ATL, pMTG) exhibiting a marked preference for other over self (*F*_(1,23)_ = 42.43, *P* < 0.001, ${\eta}_{\mathrm{p}}^2$ = 0.65). Note that all of these DN and SN regions are significantly above the resting baseline. This underscores the fact that, despite different regions showing preferential reaction to AM or ToM, activities of both networks ramp up during both introspective tasks. Next, we focused on the IPL—this analysis is motivated by two threads of evidence: 1) the anterior IPL sector (i.e., the TPJ) is more closely linked with the “SN-leaning” dmPFC, whereas the posterior sector (the AG) is more linked with the “DN-leaning” vmPFC (Bzdok et al. 2013; Braga et al. 2019); 2) the bilateral TPJ has been shown to be involved in simulating the mental states of other people, with the right TPJ tending to show more pronounced responses than its left counterpart (e.g., Saxe and Kanwisher 2003; Saxe and Wexler 2005). We selectively examined the response profiles of the bilateral AG and TPJ, and the results are shown in Figure 4*D*: Although the AG and TPJ are adjacent to each other, they prefer different types of introspective activities (*F*_(1,23)_ = 93.34, *P* < 0.001, ${\eta}_{\mathrm{p}}^2$ = 0.80). The bilateral AG shows an overall preference for the self-referential AM condition over the other-referential ToM condition (*P* = 0.007, although the preference is more exaggerated in the right AG). By contrast, the TPJ shows an overall preference for ToM over AM, reliably seen in both hemispheres (*P* < 0.001). Finally, we focused on a set of regions displaying a preference for other-referential processing—the dmPFC and the bilateral TPJ (Fig. 4*E*). We specifically examined whether their response profiles alter with different types of other-referential processing: assessing the personality traits of someone (Experiment 1) versus simulating what someone might be thinking (Experiment 2). A significant interaction statistically supports their distinct characteristics (*F*_(1,23)_ = 7.75, *P* = 0.001, ${\eta}_{\mathrm{p}}^2$ = 0.25): While the response amplitude of the dmPFC does not differ between the personality knowledge and ToM contexts (*P* = 0.27), the left TPJ (*P* = 0.002) and right TPJ (*P* < 0.001) both reveal a robustly greater response to ToM compared to personality knowledge. Also see Supplementary Figures 3 and 4 for 1) a discussion about the involvement of various semantic-related regions in the personality trait task, regardless of whether the target under evaluation is self or other, and 2) an additional analysis on three middle temporal lobe (MTL) regions.

Taken together, these results depict a coherent pattern that complements our earlier findings—albeit somewhat oversimplified, an “inward versus outward” principle captures crucial aspects of the functional profile of the broad default system. Whereas DN regions prefer introspective activities that are disjoined from external events, self-referential, and episode-based (remembering), SN regions prefer activities that are other-referential and fact-based (knowing). Furthermore, our data corroborate a division of labor that has been documented previously (Wagner et al. 2012, 2016; Heleven and Van Overwalle 2018)—within the subsystem that prefers other-referential processing, the dmPFC is recruited whenever a task involves ascribing socioemotive features to a person, be it self or other, whereas the TPJ is preferentially recruited for “online” stimulation of mental states during the ToM-type of tasks.

**Figure 5:**
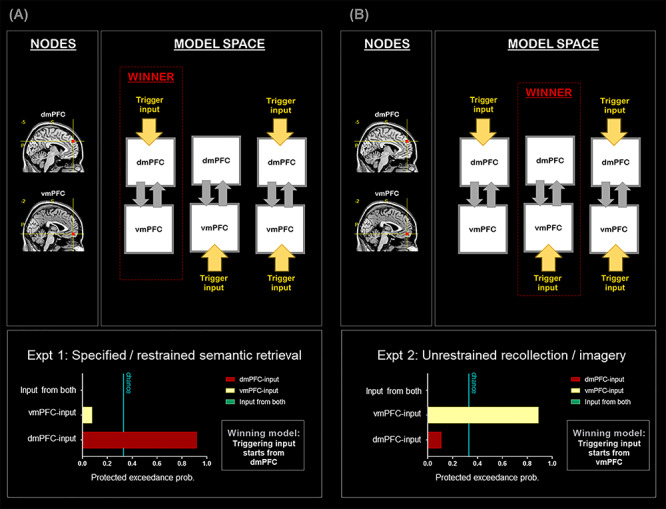
(*A*) DCM-1: All analyses are based on the data of Experiment 1. (*B*) DCM-2: All analyses are based on the data of Experiment 2. Note that the nodes of the two DCM have the same coordinates, suggesting a drastic change of network dynamics as a result of experimental contexts.

### Externally and Internally Oriented Task Focuses Modulate the Neural Dynamics of the DN and SN

We capitalized on dynamic causal modeling (DCM; Friston et al. 2003; Friston et al. 2019) to investigate whether and how the causative/directional neural dynamics within and between the DN and SN alter as a result of switching between externally and internally directed task focus. Decades of validations based on empirical fMRI data have substantiated the efficacy of DCM in making inferences about the causative connectivity between brain regions, outperforming other analytical approaches of causative connectivity (for review, see Friston et al. 2013). Particularly, DCM enables researchers to apply a Bayesian statistical procedure to compare and select, among multiple candidate models, the “winner” model that offers the most probable explanation regarding the mechanistic neural implementation that generates the observed fMRI data (Stephan et al. 2009; Rigoux et al. 2014). This statistical procedure estimates the “protected exceedance probability” (Rigoux et al. 2014), quantifying whether a model’s explanatory power surpasses all other candidates included in the space of comparison, above and beyond the likelihood of all models being equiprobable (i.e., the chance level for a given model space). Here we report a series of DCM analyses, investigating whether the directionality of communication between/within the DN and SN changes depending on whether the task encourages mental activities that are more externally oriented (Experiment 1: The personality trait tasks that confine thoughts, require explicit motoric response, and stipulate stimulus-response mappings) versus internally oriented (Experiment 2: The AM and ToM tasks that allow unrestrained thoughts and do not entail any overt response). As discussed below, for different models we assumed that incoming signals enter the network through a different brain area of the DN or SN, which represents the “inception” event that triggers subsequent neural dynamics via different routes. We then employed Bayesian statistical methods to identify the best model that underlies the fMRI data.

**Figure 6:**
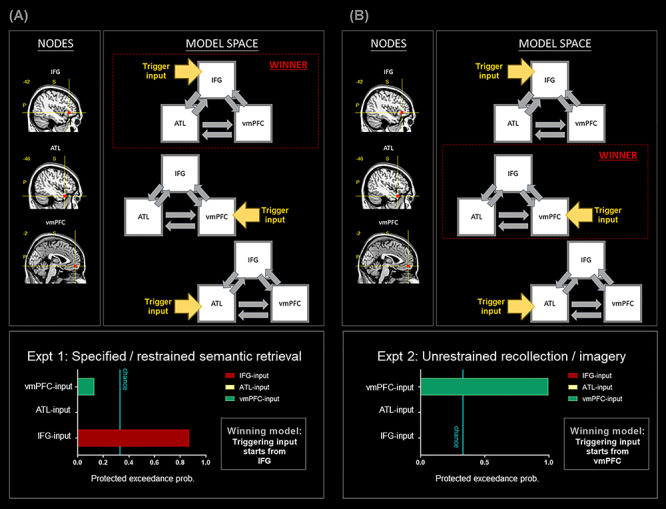
(*A*) DCM-3: All analyses are based on the data of Experiment 1. (B) DCM-4: All analyses are based on the data of Experiment 2. Note that the nodes of the two DCM have the same coordinates, suggesting a drastic change of network dynamics as a result of experimental contexts.

The first (Experiment 1; Fig. 5*A*) and second (Experiment 2; Fig. 5*B*) DCM analyses were focused on the mPFC. Our earlier analysis reveals a local-scale bipartite split, with the dmPFC favoring externally directed tasks and the vmPFC favoring internally directed tasks. Based on this, we examined whether task contexts impact on the “starting point” from which the flow of neural processing cascades downstream. This was implemented by assuming, for different models, a different “entry” location through which the triggering input enters the two-node network (dmPFC vs. vmPFC). For each DCM, we constructed three models, hypothesizing that the trigger enters the system through the dmPFC, vmPFC, or both nodes (see Fig. 5*A*,*B*). Exactly identical coordinates of the dmPFC and vmPFC were applied to localize the network nodes for the analyses of Experiments 1 and 2. This permits a rigorous test on whether the very same group of brain regions adjust their interplay under externally versus internally veered contexts, through the comparison of the two DCM outcomes. Using well-established Bayesian procedures (Daunizeau et al. 2014; Rigoux et al. 2014) to select the model that provides the best mechanistic explanation for the fMRI data, we found that task focus drastically changes the directionality of neural dynamics. As clearly indicated by the magnitude of protected exceedance probabilities, when the task was focused on externally directed mental activities (Experiment 1; Fig. 5*A*), the dmPFC-input model overwhelmingly outperformed other models and was the only model that substantially exceeded the chance level. By contrast, when the task encouraged internally directed processes (Experiment 2; Fig. 5*B*), the vmPFC-input model became the winner that strikingly outperformed other models and was the only model that performed above the chance level. This demonstrates the reversal effect of task contexts—when the task was directed externally, the SN-leaning dmPFC initiated the onset of neural dynamics; by contrast, when the task was directed internally, the DN-leaning vmPFC became the trigger.

Next, in the third (Experiment 1; Fig. 6*A*) and fourth (Experiment 2; Fig. 6*B*) DCM, we focused on the trilateral communication among the IFG, the ATL, and the vmPFC. These rostral–frontotemporal nodes were included in the model owing to their respective roles in controlling semantic retrieval (the IFG: Noonan et al. 2013; Chiou et al. 2018), representing semantic concepts (the ATL: Lambon Ralph et al. 2017), and underpinning internally focused thoughts (the vmPFC: Spreng and Grady 2010). We constructed three models for each DCM, assumed a different starting point for each model (IFG-input, ATL-input, or vmPFC-input), and set both DCM to be based on identical nodes. Results of Bayesian model selection are shown in Figure 6: During the externally focused Experiment 1, the IFG-input model exceedingly outperformed other models and was the only model that surpassed the chance level (Fig. 6*A*). However, during the internally focused Experiment 2 (Fig. 6*B*), the network changed the dynamics between nodes—the vmPFC-model became the one that won over other models by a massive margin, offering the best account for the underlying neural interactions. This yields a consistent pattern across analyses—under an externally biased context, neural dynamics stemmed from a SN site (DCM-1, DCM-3), whereas under an internally biased situation, neural dynamics commenced from a DN site (DCM-2, DCM-4).

**Figure 7:**
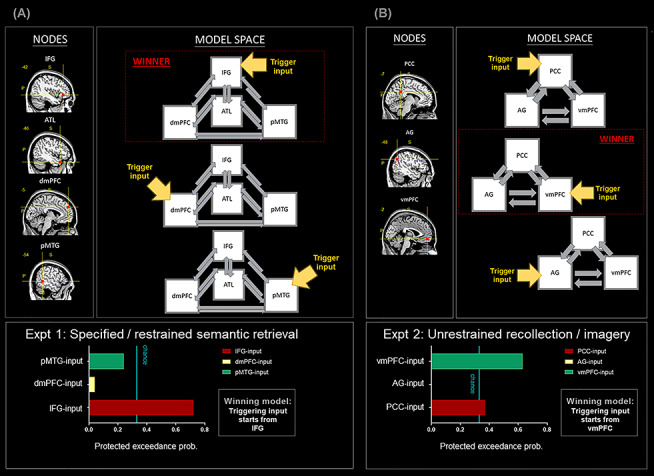
(*A*) DCM-5: All analyses are based on the data of Experiment 1. All of the nodes belong to the SN. (*B*) DCM-6: All analyses are based on the data of Experiment 2. All of the nodes belong to the DN.

Finally, in the fifth (Experiment 1; Fig. 7*A*) and sixth (Experiment 2; Fig. 7*B*) DCM, we specifically focused on the SN for Experiment 1 and on the DN for Experiment 2, motivated by the robust contextual modulations that we observed in earlier analyses. These analyses aimed to identify, among all constituent sites within the SN/DC, the most reliable region that launches neural dynamics under an externally and internally biased context. Thus, in DCM-5, we included all the key nodes closely associated with the SN—the IFG, the ATL, the pMTG, and the dmPFC. We constructed three models, hypothesizing the triggering signal might start from the IFG, the pMTG, or the dmPFC. Results of Bayesian model selection showed that, as illustrated in Figure 7*A*, the IFG-input model gained the highest protected exceedance probability and was the only model above the chance threshold, trumping other models. By contrast, in DCM-6, we included all the core nodes of the DN—the vmPFC, the PCC, the AG. Three models were built, with the triggering signal entering via the vmPFC, the PCC, or the AG. As shown in Figure 7*B*, results of model comparison indicated that the vmPFC-input model gained most Bayes posterior likelihood, greatly outperforming other hypotheses. Taken together, this series of DCM analyses clearly demonstrated the fluidity of context-dependent neural dynamics—akin to a tug-of-war occurring at the neural level, an externally focused task propels information to flow from a SN origin to the DN (with the IFG being the most reliable triggering point), whereas an internally focused task drives signal to travel from a DN origin to the SN (with the vmPFC being the most reliable triggering unit).

## Discussion

In the present study, we conducted a series of analyses to investigate the fusion and fission between the DN and SN under various contexts. Results revealed a highly robust dissociation—within the distributed network that favors internally constructed representations and tends to shy away from sensory-motoric processes, we found that each region’s preferential reactions to the subtle distinction between contexts lead to a bipartite subdivision within the network. One subset of regions constitute the DN—it shows a strong antipathy for externally directed visuospatial tasks, tends to withdraw from tasks that require attention to external entities (e.g., reading text, pushing buttons), and prefers self-referential to other-referential processing even when the psychophysical settings of tasks are yoked. Another subset of regions constitute the SN—its aversive response to visuospatial tasks is more moderate than that of the DN; it actively participates in various sociosemantic tasks unaffected by the need to attend to external entities (unlike the DN that shows minimal involvement under such circumstances), and it prefers other-referential to self-referential processing. Furthermore, a task context that encourages participants to focus inwardly drives neural dynamics to arise from the DN (with the vmPFC being the most robust initiating region), whereas a context that requires an outward focus drives neural dynamics to emanate from the SN (with the IFG being the most reliable leadoff area). Taken together, the clearly distinct functional profiles of the DN and SN suggest that, while the two networks similarly show a distaste for tasks requiring nonmeaningful sensory-motoric processing, they diverge along an “internal versus external” neurocognitive dimension, with the DN being more inwardly biased and the SN more outwardly biased. Below we discuss our findings under the framework of recent proposals concerning a macroscale gradient that span across the entire cerebral zones.

### The Evolution of Our Definition of the Broad Default System

As discussed above, early hypotheses discredit the potential function that the broad DN serves in goal-directed behavior and instead postulate that it is associated with off-task mind-wandering and lapses during a task (e.g., Raichle et al. 2001). Such “task-negative” or “hindrance” views have been challenged by the robust observations that many goal-driven introspective processes positively engage various regions of the traditional “task-negative” network to differential degrees (e.g., Spreng and Grady 2010). Recent findings have further shown that the extent of elaborated details that one maintains in working memory, be it visuospatial or semantic in nature, is coded by the multivoxel pattern of various DN and SN regions (Sormaz et al. 2018; Turnbull et al. 2019) and that the activity of both DN and SN ramps up when a task becomes less reliant upon perceptual input and more upon contents of working memory (even when the content is purely visuospatial, although semantic content elicits greater activity; Murphy et al. 2018, 2019a). These results have led to the contemporary accounts that the brain is equipped with adaptive machinery, implemented by the DN and SN, that supports cognition when it has to rely on internally constructed representations and external stimuli are useless or unavailable (Wang et al. 2020). Care should be taken when interpreting the novel evidence about the DN/SN’s role in supporting the working memory of visuospatial stimuli, given the fact that the choice of baseline affects the presence and absence of DN/SN activation. While a direct contrast of one-back visual working memory against visual perception reveals DN/SN activation (e.g., Murphy et al. 2019b), the system might become deactivated 1) when one-back working memory is compared against resting periods (owing to detailed inner thoughts more probable to arise during rest; see Visser et al. 2010) or 2) when strenuous two- or three-back working memory is pitted against effortless zero-back/visual perception (i.e., the regular observation of difficulty-induced deactivation; Anticevic et al. 2010). A challenge for future research is to clarify how multiple determinants (internal/external focus, task difficulty, extent of detailed contents) jointly modulate the activity of DN/SN. Taken together, the field’s continually evolving definition of the system signifies the significant progress made. In this context, our current findings reveal various situations wherein the DN and SN have differential preferences, providing important clues about the system’s accurate characterization.

### Large-Scale Gradational Structure of the Human Cortex

Recent advances in mapping the human cortex have discovered a clear gradient of structural and functional features that spans across the sensory-motoric system and the multimodal system, found in both humans and nonhuman primates (Margulies et al. 2016; Vidaurre et al. 2017; Huntenburg et al. 2018; Dixon et al. 2018; Kernbach et al. 2018; Oligschläger et al. 2019). An evident neurocognitive dimension of “internal versus external preference” underlies this cortical gradient—on one extreme of the spectrum, the cortical regions are highly modality-specific (e.g., the primary visual/auditory cortex; Margulies et al. 2016), respond to external events occurring here and now (i.e., showing narrow spatiotemporal receptive fields; Baldassano et al. 2017), and prefer concrete, perceivable stimuli than abstracted, conceptual stimuli (de Heer et al. 2017). Regions on the other extreme of the spectrum (e.g., the core DN areas; Margulies et al. 2016), on the contrary, exhibit the opposite characteristics—multimodal, able to represent information across long spatial and temporal extents, and sensitive to conceptual-mnemonic stimuli. Juxtaposed between the polar extremes are multiple intermediate zones, such as the SN that leans towards the DN extreme, the dorsal attention system that leans towards the sensory-motoric extreme (Dixon et al. 2017), and the frontoparietal control system that is situated midway between the two ends (Spreng et al. 2010). Within the frontoparietal control system, recent evidence has demonstrated that this middle ground further fractionates into two sectors: One subsystem preferentially couples with the attention and sensorimotor systems,and the other subsystem preferentially links with the DN and SN (Dixon et al. 2018). A similar bipartite split was also found in the dorsal attention network (de Heer et al. 2017). In the present study, we provide crucial evidence that delineates the fine-grained fractionation within the high-level cerebral territory by revealing the commonalities and, more importantly, the striking distinctions in the functional profiles of the DN and SN. Our findings echo with recent evidence that, while the exact spatial arrangement of different functional networks is configured in an idiosyncratic, locally interdigitated manner in each individual’s brain, a clear “motif” (*cf.*, Braga and Buckner 2017; Braga et al. 2019) of bipartite dissociation is consistently observed in multiple networks, producing a gradational tapestry-like structure across the entire brain (Braga and Buckner 2017; Braga et al. 2019; DiNicola et al. 2020).

### Underpinning of the Gradient Structure

Various hypotheses have been proposed regarding the mechanisms that drive the formation of the cortical gradient of the human brain. One potential root cause might be the cytoarchitecture and myeloarchitecture of the cortical sheet (for review, see Huntenburg et al. 2018). For instance, it has been shown that cortices of the sensory-motoric system contain higher numbers of neurons but relatively fewer synaptic connections between cells, whereas the multimodal system (e.g., the DN or prefrontal cortex) contains fewer neurons but more synapses (Collins et al. 2010). The level of myelination also differs; sensory-motoric regions are more myelinated (hence, permitting swift conduction of information flow and prompt response to external stimuli), whereas higher-order regions are less myelinated and requires longer time to process (Huntenburg et al. 2017). In addition, studies based on diffusion tractography (Binney et al. 2012; Bajada et al. 2019) and large-scale connectome database (de Wael et al. 2018) have coherently identified a profile of graded connectivity that the SN and DN are transmodal zones where multiple circuitries progressively converge, receiving inputs from various primary sensory and intermediate zones. Such structural–anatomical evidence suggests a possibility that, although many regions of the SN are often incorporated into the canonical umbrella term “default-mode system,” these regions might have different cyto- and myelostructures and connectivity profiles, which make the SN behave differently from the core regions of the DN on a variety of tasks as we demonstrate here. One possibility could be that, while both the DN and SN are situated on the multimodal end of the gradient, there is relatively shorter “distance” (in terms of the length of graph metric) from the SN to the sensory-motoric cortices. This could make the SN more responsive to external sensory stimuli, compared to the DN that has longer, more convoluted access to sensory cortices. This speculation awaits future investigation.

### Integration between Semantic Cognition with Default-Mode Processes

Brain regions that support different aspects of semantic cognition have long been studied separately from the regions engaged during resting state. Given the fact that default-state processes recruit substantially overlapped areas with semantic cognition (Binder et al. 2009) and that the DN and SN are closely connected (Humphreys et al. 2015; Jackson et al. 2016), more cross talk between the two bodies of literature is necessary. Under the framework of a macroscale cortical gradient, the DN and SN could be construed as two complementary systems that work in tandem to serve high-level cognition. The DN areas have been shown to be maximally distant from the sensory-motoric cortices (Margulies et al. 2016). Its locus explains why the DN sits atop the information processing hierarchy and represents most abstract forms of thoughts divorced from different input modalities (e.g., social relationships or self-related contemplation). In contrast, the location of the SN allows it to interface between the DN, the frontoparietal control system, and the sensory-motoric system—which is critical to controlled semantic cognition (Lambon Ralph et al. 2017). The formation of generalizable, coherent concepts is known to rely upon the ATL, a transmodal representational hub that can interface with all verbal and sensory-motoric modalities simultaneously (Lambon Ralph et al. 2017; Chiou and Lambon Ralph 2019). In addition, for controlled semantic processing, the semantic representational system needs to interface with executive control mechanisms, underpinned by the IFG and pMTG, in order to generate contextually appropriate behaviors (Chiou et al. 2018). Presumably, the DN and SN work together when the context entails goal-directed cognitive operation that combines self-referential processing with semantic knowledge, as in the retrieval of autobiographical memory.

## Conclusion

In the present study, we report a series of fMRI findings that manifest a clear-cut bipartite split in the functional profiles between the DN and SN, evident both in their response amplitudes to different situations and neural dynamics. These discoveries are consistent with recent theories regarding a macroscale cortical gradient that encompasses the entire cerebrum. The bipartite split on multiple inter-related functional dimensions (inwardly directed, self-referential, abstract, multimodal, disengaged from the immediate and perceptible environment *vs.* outwardly directed, other-referential, concrete, unimodal, engaged in the immediate and perceptible stimuli) indicates the crucial need that understanding the relationship between the DN and SN, as well as their relationship with other functional networks, requires more thorough understanding about the macroscale architecture of the human brain.

## Funding

MRC program (grant MR/R023883/1 to M.A.L.R.); Sir Henry Wellcome Fellowship (201381/Z/16/Z to R.C.).

*Conflict of Interest*: The authors declare no competing financial interests.

## Supplementary Material

Supplemental_information_AcceptedVersion_bhaa130
