# Supplementary material for: Bipartite Functional Fractionation within the Default Network Supports Disparate Forms of Internally Oriented Cognition
Source: Cereb Cortex. 2020 Jun 4;30(10):5484–501. doi: 10.1093/cercor/bhaa130 (PMC7472201; doi:10.1093/cercor/bhaa130)
Supplement: Supplemental_information_AcceptedVersion_bhaa130 [file supplemental_information_acceptedversion_bhaa130.pdf]

Supplemental information:

Bipartite functional fractionation within the default network supports disparate forms of internally oriented cognition

Rocco Chiou✉, Gina F. Humphreys, & Matthew A. Lambon Ralph✉
MRC Cognition and Brain Sciences Unit, University of Cambridge, UK

✉ Correspondence to this work can be addressed to either of the authors by email:
(**RC:** Rocco.Chiou@mrc-cbu.cam.ac.uk, **MALR:** Matt.Lambon-Ralph@mrc-cbu.cam.ac.uk)

Table of Contents

Supplemental Methods **2**

Supplemental Figures and Discussions **6**

Supplemental References **14**

Supplemental Methods

MRI acquisition. All scans were acquired using a 3T Phillips Achieva scanner equipped with a 32-channel head coil and a SENSE factor of 2.5. A dual-echo EPI sequence was used to maximise signal-to-noise ratio in the rostral-ventral surface of the brain that has been known to susceptible to signal dropout (Halai *et al.*, 2014). Using this technique, each scan consisted of two images acquired simultaneously with different echo times: a short echo optimised to obtain maximum signal from the ventral parts and a long echo optimised for whole-brain coverage. The sequence included 31 slices covering the whole brain with repetition time (TR) = 2.8 sec, short / long echo times (TE) = 12 / 35 ms, flip angle = 85°, field of view (FOV) = 240 × 240 mm, resolution matrix = 80 × 80, slice thickness = 4 mm, and voxel dimension = 3 × 3 mm on the *x*- and *y*-axis. To reduce ghosting artefacts in the temporal lobes, all functional scans were acquired using a tilted angle, upward 45° off the AC-PC line. Functional scans of the two main experiments were collected over seven runs; each run was 432-sec long during which 155 dynamic scans were acquired (alongside 2 dummy scans, discarded). To tackle field-inhomogeneity, a B_0 field-map was acquired using identical parameters to the functional scans except for the following: TR = 599 ms, short / long TEs = 5.19 / 6.65ms. Total B0 scan time was 1.6 minutes. A high-resolution T_1-weighted structural scan was acquired for spatial normalisation, including 260 slices covering the whole brain with TR = 8.4 ms, TE = 3.9 ms, flip angle = 8°, FOV = 240 × 191 mm, resolution matrix = 256 × 163, and voxel size = 0.9 × 1.7 × 0.9 mm. Total structural scan time took 8.19 minutes.

Pre-processing. Analysis was carried out using SPM8 (Wellcome Department of Imaging Neuroscience). The functional images from the short and long echoes were integrated using a customised procedure of linear summation (Halai *et al.*, 2014; Poser *et al.*, 2006). The combined images were realigned using rigid body transformation (correction for motion-induced artefacts) and un-warped using B0 field-map (correction for field-inhomogeneity). The averaged functional images were then co-registered to each participant's T_1 anatomical scan. Spatial normalisation into the MNI standardised space was achieved using the DARTEL Toolbox of SPM (Ashburner, 2007), which has been shown to produce highly accurate inter-subject alignment (Klein *et al.*, 2009). Specifically, the T_1-weighted image of each subject was partitioned into grey-matter, white-matter, and CSF tissues using SPM8's 'Segmentation' function; afterwards, the DARTEL toolbox was used to create a group template derived from all participants. The grey-matter component of this template was registered into the SPM grey-matter probability map (in the standard MNI stereotactic space)

using affine transformation. In the process of creating the group's template brain using individual T_1, for each individual DARTEL estimated 'flow fields' that contained the parameters for contorting native T_1-weighted images to the group template. SPM8 deformation utility was then applied to combine group-to-MNI affine parameters with each participant's 'flow fields' to enable tailored warping into the MNI space with better accuracy. The functional images were then resampled to a 3 × 3 × 3 mm voxel size. Smoothing was subsequently applied using an 8-mm Gaussian FWHM kernel, consistent with prior studies (e.g., Halai *et al.*, 2014; Jackson *et al.*, 2015).

GLM analysis. For each participant, contrasts of interest were estimated using general linear model (GLM) convolving the experimental design matrices with a canonical haemodynamic response function, with resting periods modelled implicitly. Motion parameters were entered into the model as covariates of non-interest. For Experiment 2, we separately modelled the events of interest (i.e., the 15-sec interval of AM, ToM, and VS) and the button-response interval at the end so that the results are not contaminated by response preparation or execution. Moreover, we included each participant's reaction times (RTs) of all active-task performance as parametric modulators, allowing us to rule out any brain activation driven by task difficulty or cognitive effort when assessing the effects of experimental manipulation. Low-frequency drifts were removed using a high-pass filter of 128 sec. Contrast images from the individual-level (1st-level) analyses were then submitted to random-effect models in the group-level (2nd-level) analyses.

<u>Coordinates of the regions of interest (ROIs)</u>. The table below displays all of the ROIs used in the present study, their coordinates in the MNI stereotaxic space, and location pinpointed on the MNI template by the yellow crosshair. Also rendered on the template is the meta-analysis outcomes, based on the NeuroSynth database, of brain regions related to default-mode network and semantic memory.

ROI	MNI-coordinate	Location
dmPFC	-5, 54, 34	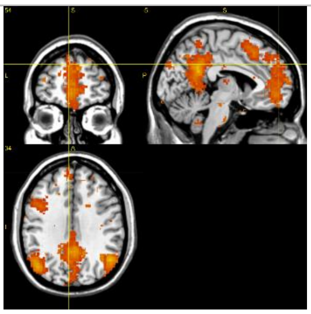
vmPFC	-2, 54, -12	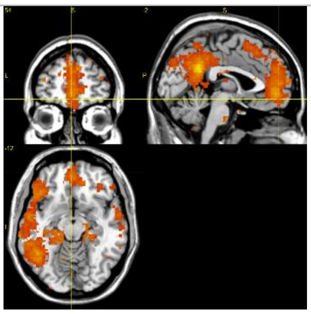
PCC	-7, -48, 31	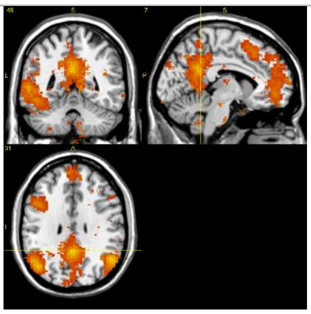
L.AG	-48, -64, 34	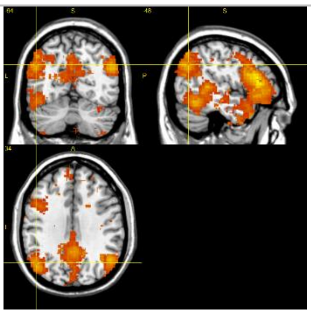
R.AG	48, -64, 34	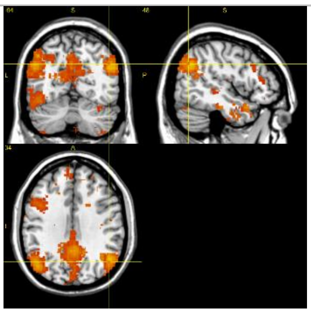

ATL	-46, 18, -26	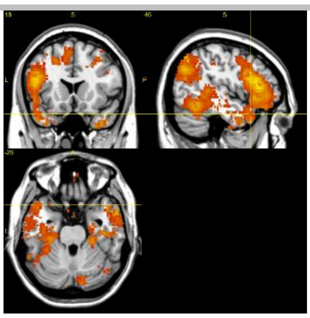
IFG	-42, 34, -6	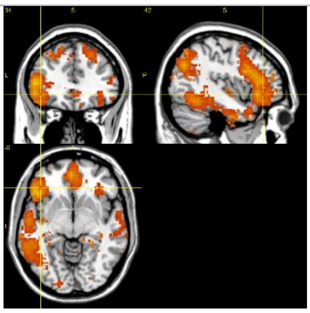
pMTG	-54, -49, -1	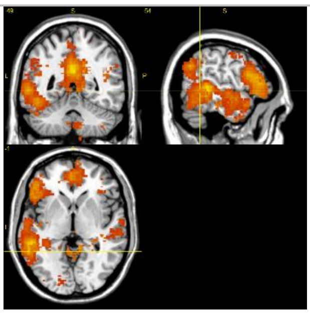
L.TPJ	-57, -42, 20	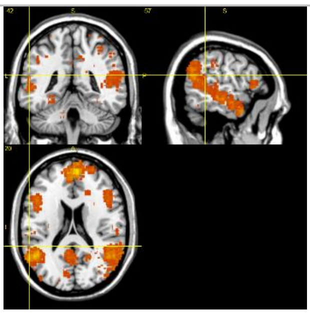
R.TPJ	57, -42, 20	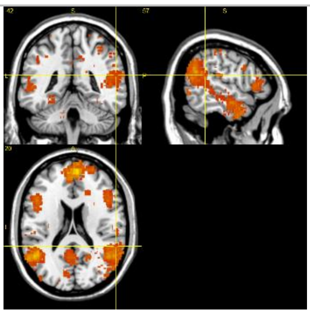

Supplemental Figures and Discussions

Behavioural analysis. In Experiment 1, participants showed comparable reaction times for the Self- (average±SD: 1071±35 ms) and Other-Referential (1056±39 ms) tasks, with no statistically reliable difference between them (paired *t*-test, $p = 0.42$). In Experiment 2, participants also showed comparable reaction times for the AM (807±36 ms) and ToM (832±44 ms) conditions, with them having no reliable difference ($p = 0.39$; note that these reaction times represent the latency to rate vividness *after*, rather than during, an AM- or ToM-interval). More importantly, the results of vividness ratings indicated that participants were able to engage in highly vivid recall and imagery (AM: 1.25±0.04; ToM: 1.28±0.05, both approached the ceiling-level on the given scale), while the ratings did not differ between contexts ($p = 0.64$). Together, these data showed that, in both experiments, the tasks specifically designed to probe the functionality of the default network are matched on the general cognitive effort required (as indicated by reaction times) and the clarity of introspective experiences (as indicated by the vividness ratings). Also note that in all fMRI analysis, reaction times (including those of the visuospatial tasks) were included as additional regressors to factor out their influences on the neural data.

Supplemental Figure S1.

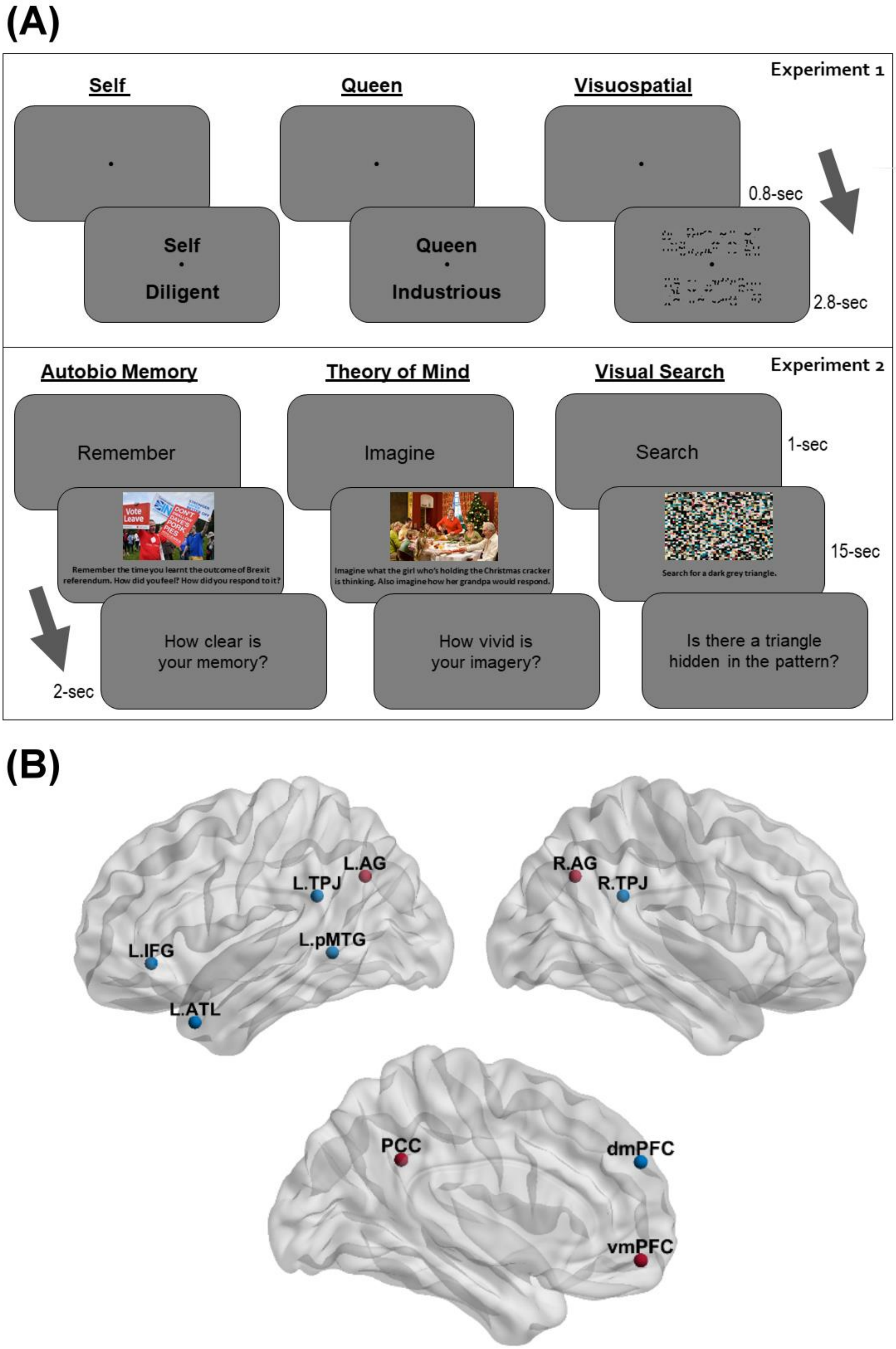

Figure S1. **(A)** Experiment 1 has three task-conditions: Self-Concept (assessing if the adjective fits one's own personality traits), Other-Concept (assessing if it fits the participant's impression for the Queen), Visuospatial (answering if the scrambled patterns are mirror-reversed images); Experiment 2 also includes three task-conditions: Autobiographical Memory (recalling life events related to the topic), Theory of Mind (imagining the feelings and thoughts of the person in the photograph), and Visual Search (looking for a tiny triangle); both Experiment 1 and 2 includes resting-state periods that are randomly interleaved with the task-conditions. **(B)** The locations of the ten ROIs in our analysis are rendered on a glass brain that highlights their relative position. Colour coding: red indicates an ROI that tends to be affiliated with the core default network; blue indicates an ROI that tends to be affiliated with the semantic network.

Supplemental Figure S2.

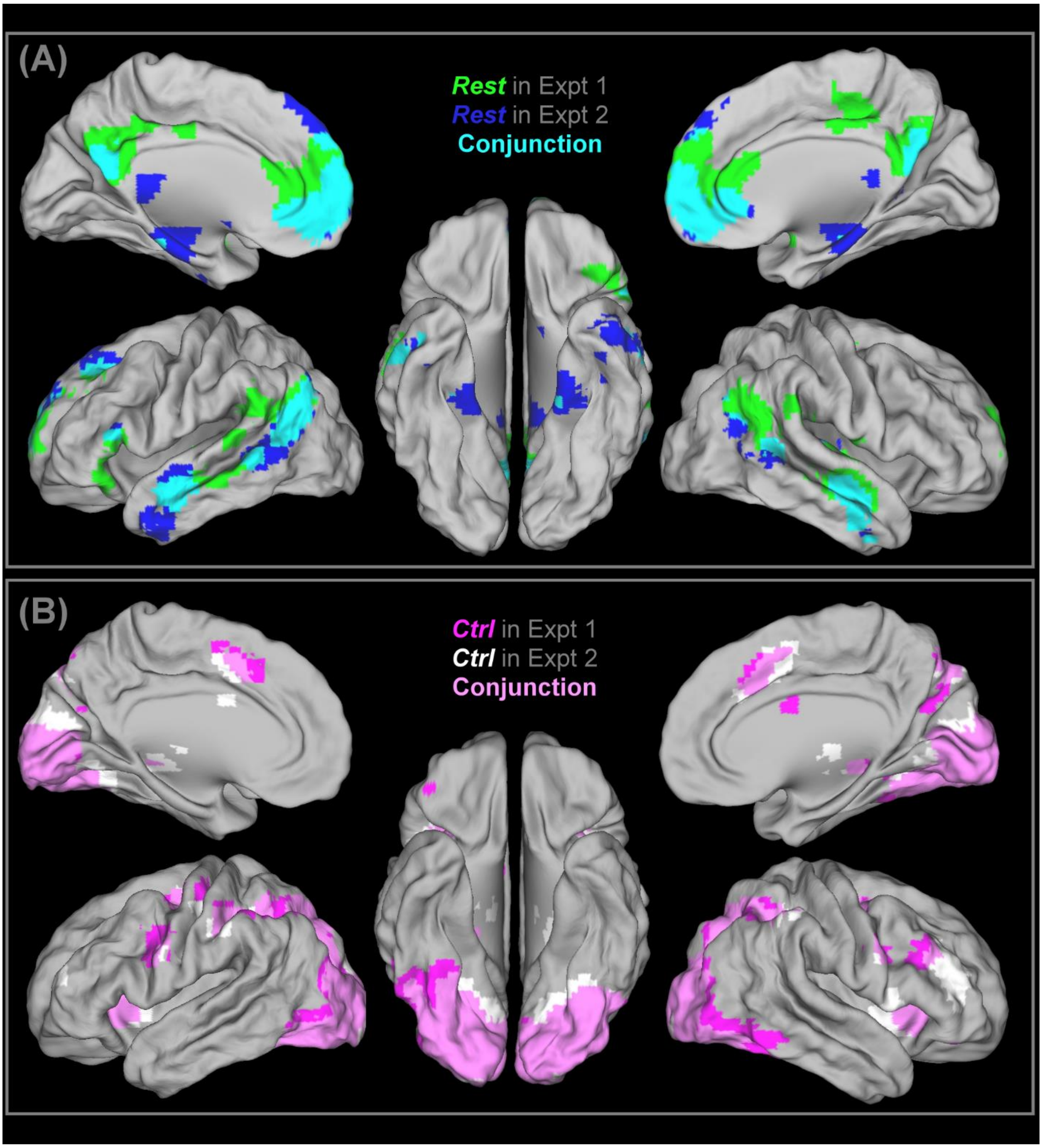

Figure S2.

(A) Resting-state activities (Rest > Control) during Experiment 1 and Experiment 2 are represented using green and blue, respectively; the areas of conjunction is cyan. As discussed in the main text, key regions of the broad 'task-negative' network are reliably involved during rest in both experiments (i.e., the cyan areas that indicate overlaps). These include the core default network (DN): the medial prefrontal cortex (mPFC), the posterior cingulate cortex and retrosplenial cortex (PCC and RSC), and the angular gyrus (AG); the semantic network (SN): the lateral anterior temporal lobe (ATL), the inferior frontal gyrus (IFG), and the posterior mid-temporal gyrus (pMTG); there is discrepancy between Experiment 1 and Experiment 2 in the 'diagnostic' regions that dissociate 'Network-A' and 'Network-B' (Braga & Buckner, 2017) at the dorsal PCC, the parahippocampal cortex (PHC), the anterior sector of the inferior parietal lobule (conventionally called the temporoparietal junction/TPJ). We discuss the potential driving factor that causes this discrepancy in the main article.

(B) Task-driven activities (Control > Rest) of Experiment 1 and Experiment 2 are represented using magenta and white, respectively; the areas of conjunction is pink. We observed similar patterns of neural activity driven by the mental rotation task (Experiment 1) and the visual search task (Experiment 2). These two visuospatial tasks activated a set of widely distributed 'task-positive' regions or the 'multiple-demand network' known to underpin spatial attention and executive control. As illustrated in Fig. *S*2, both tasks elicited robust activity of the frontal and parietal cortices, also the anterior cingulate cortex, that are known to support attention, executive control, and visuospatial working memory (for review, see Fedorenko, Duncan, & Kanwisher, 2013; Nee *et al.*, 2013), as well as the bilateral visual cortices.

Supplemental Figure S3.

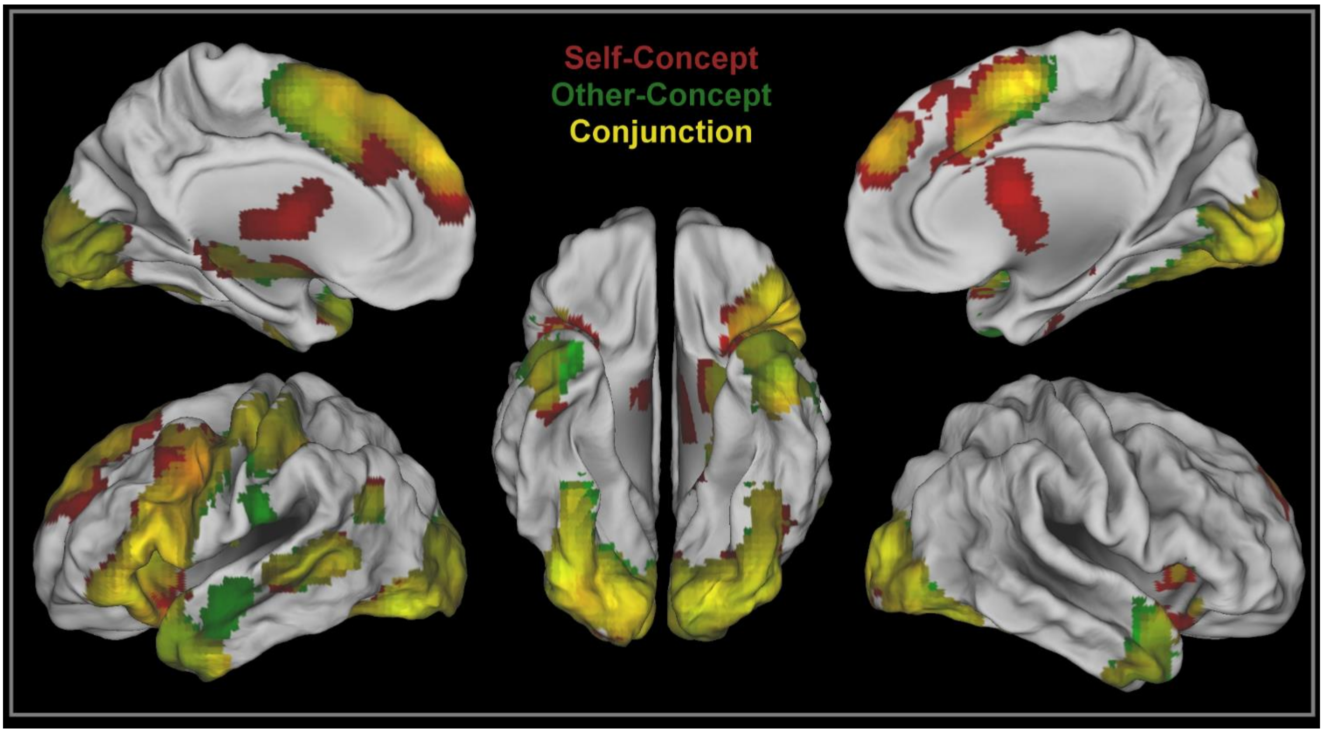

Figure S3. Task-driven activities during Experiment 1. Red represents activation driven by the '*Self-Concept* > *Rest*' contrast. Green represents the results of '*Other-Concept* > *Rest*' contrast. Yellow represents the areas of conjunction. There are three main findings here: (***i***) Self-Concept and Other-Concept both activate extensive swathes of the semantic system, with substantial overlaps; all of the key regions of the semantic network are involved in both conditions – the ATL, the left IFG, the left pMTG, and the dorsal section of medial prefrontal cortex (dmPFC). This highlights the fact that the semantic network is generally involved in evaluating semantic information (in the present case – semantic information about personality traits), regardless of whether the person under consideration is self or another individual. (***ii***) As discussed in the main article, core nodes of the default network – the ventral section of the medial prefrontal cortex (vmPFC), the PCC, and the AG – are less involved in this more externally-oriented task of personality assessment, as indicated by the absence of active cluster at these sites. This is in striking contrast with their robust activity in the internally-oriented task-settings during Experiment 2 (lengthy intervals during which participants were immersed in their internal thoughts). (***iii***) Finally, as a sanity check – there is robust activity in these task-conditions in the bilateral visual cortices (as the stimuli were presented visually) and in the left sensorimotor cortex (as participants were responding using their right hand), indicating that the analysis are performed accurately that gives the correct neural signatures that ought to appear as a result of our experimental design.

Supplemental Figure S4.

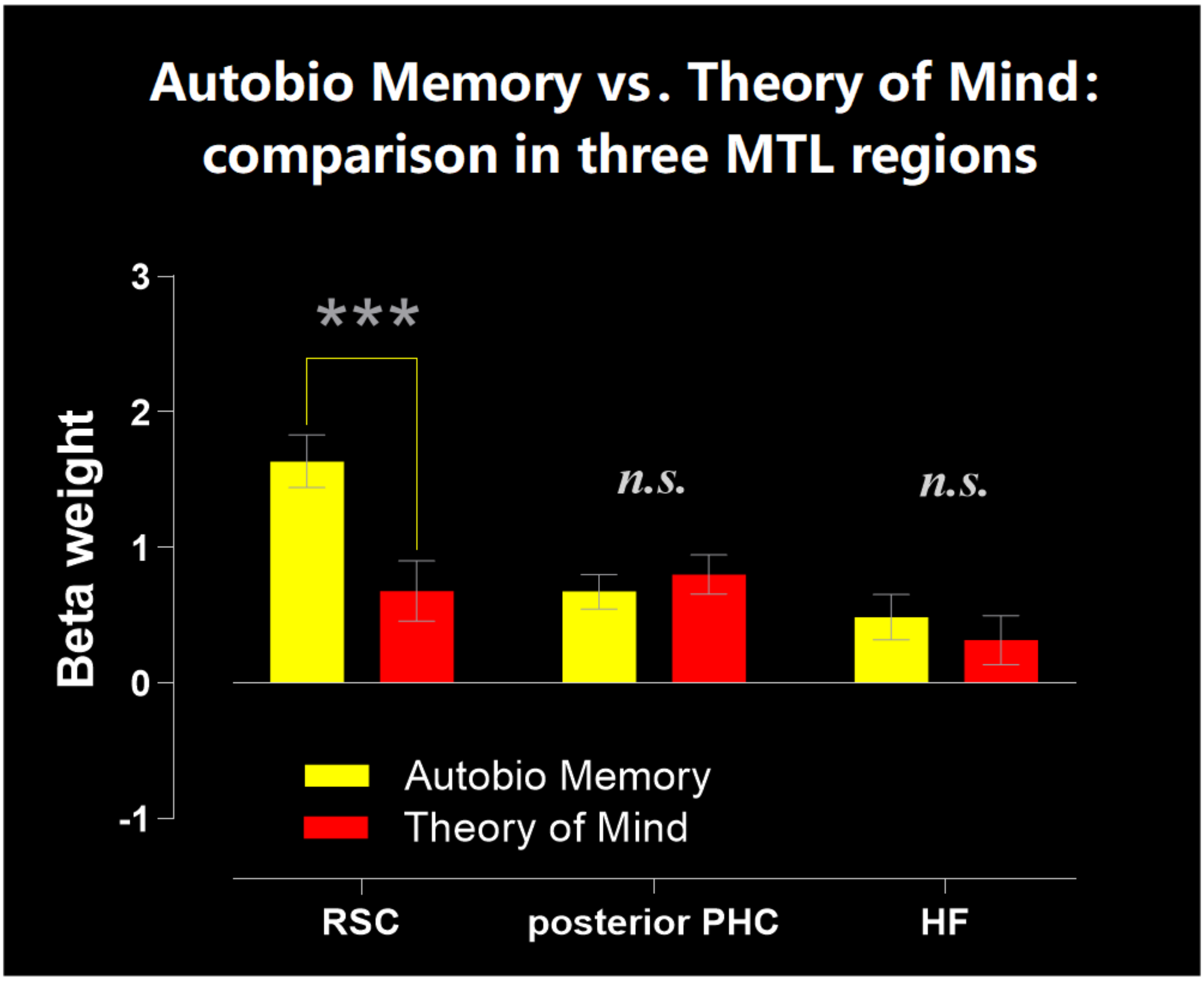

Figure S4. In this analysis we specifically focus on the medial temporal lobe (MTL), a neural structure known to be involved in a wide range of internally-directed contexts, such as autobiographical memory, resting-state cognition, episodic-retrieval processes, etc. We select three ROIs based on the peak coordinates of an influential study by Buckner and colleagues (Andrews-Hanna *et al.*, 2010): the retrosplenial cortex/RSC: [-14, -52, 8]; the posterior parahippocampal cortex/PHC: [-28, -40, -12]; the hippocampal formation/HF: [-22, -20, -26]. We examine the neural responses (β-weights contrasted against the resting-state, like the approach used for the analysis in the main article) and compare between the task-conditions of autobiographical memory (AM) and theory of mind (ToM). Results reveal a statistically significant interaction of the three ROIs (RSC, PHC, and HF) × the two tasks (AM *vs*. ToM), $F_{(1,23)} = 27.21$, $p < 0.001$, $\eta_p^2 = 0.54$. To identify the source of the interaction, we perform post-hoc pair-wise comparisons. As illustrated in Figure *S*4, we found that neural responses to the AM and ToM conditions do not differ in the PHC and HF (both ps > 0.14). However, there is a significant difference in the RSC ($p < 0.001$), with RSC activation for the AM task being significantly greater than the ToM task. It is also evident in the figure that responses to both tasks are greater than the resting-baseline in every ROI. These results are consistent with the previous literature regarding the role of MTL in various introspective processes. It also underlines the role of the RSC in autobiographical memory – because the AM task that we use encourages topic-guided recollection of life experiences (e.g., Recall how you felt when you just learnt the result of the Brexit referendum. Were you exhilarated or disappointed? What did you say to people about it?). This is a highly semantically loaded process (in the example here, understanding the meaning of referendum, exhilaration, and disappointment is essential). This may be particularly reliant on high-order mnemonic regions like the RSC that facilitates the creation of a meaningful mental scenario with combinations of relevant times, places, people, and sentiments.

Supplemental References

Andrews-Hanna, Reidler, Sepulcre, Poulin, & Buckner. (2010). Functional-anatomic fractionation of the brain's default network. *Neuron, 65*(4), 550-562.

Ashburner. (2007). A fast diffeomorphic image registration algorithm. *Neuroimage, 38*(1), 95-113.

Braga, & Buckner. (2017). Parallel interdigitated distributed networks within the individual estimated by intrinsic functional connectivity. *Neuron, 95*(2), 457-471. e455.

Fedorenko, Duncan, & Kanwisher. (2013). Broad domain generality in focal regions of frontal and parietal cortex. *Proceedings of the National Academy of Sciences, 110*(41), 16616-16621.

Halai, Welbourne, Embleton, & Parkes. (2014). A comparison of dual gradient-echo and spin-echo fMRI of the inferior temporal lobe. *Human Brain Mapping, 35*, 4118-4128.

Jackson, Hoffman, Pobric, & Lambon Ralph. (2015). The Nature and Neural Correlates of Semantic Association versus Conceptual Similarity. *Cerebral cortex, 25*(11), bhv003.

Klein, Andersson, Ardekani, Ashburner, Avants, Chiang, . . . Hellier. (2009). Evaluation of 14 nonlinear deformation algorithms applied to human brain MRI registration. *Neuroimage, 46*(3), 786-802.

Nee, Brown, Askren, Berman, Demiralp, Krawitz, & Jonides. (2013). A meta-analysis of executive components of working memory. *Cerebral cortex, 23*(2), 264-282.

Poser, Versluis, Hoogduin, & Norris. (2006). BOLD contrast sensitivity enhancement and artifact reduction with multiecho EPI: parallel-acquired inhomogeneity-desensitized fMRI. *Magnetic Resonance in Medicine, 55*(6), 1227-1235.
